# A2BAR Antagonism Mitigates Pulmonary Inflammation and Fibrosis and Restores Macrophage M1/M2 Balance in Diabetic Rats

**DOI:** 10.3390/ijms27188171

**Published:** 2026-09-14

**Authors:** Ángelo Torres-Arévalo, Sarah Montoya-Muñoz, Mateo Montes-Vanegas, Claudio Cappelli-León, Ignacio Árias, Claudia Jara Cancino, Claudia Quezada, Sebastián Alarcón, Benjamín Guerrero, Pamela Silva, Víctor Pola-Véliz, Rody San Martín

**Affiliations:** 1 Escuela de Medicina Veterinaria, Facultad de Recursos Naturales y Medicina Veterinaria, Sede Talca, Universidad Santo Tomás, Talca 347-3620, Chile; mateomontes.g.123@gmail.com; 2 Facultad de Salud, Institución Universitaria Colegio Mayor de Antioquia, Medellín 050036, Colombia; sarahm@est.colmayor.edu.co; 3 Laboratorio de Inmunoepigenética, Instituto de Bioquímica y Microbiología, Universidad Austral de Chile, Valdivia 511-0566, Chile; claudio.cappelli@uach.cl (C.C.-L.); ignacio.arias@alumnos.uach.cl (I.Á.); 4 Laboratorio de Patología Molecular, Instituto de Bioquímica y Microbiología, Universidad Austral de Chile, Valdivia 511-0566, Chile; claudiajaracancino@gmail.com; 5 Tumor Biology Laboratory, Institute of Biochemistry and Microbiology, Faculty of Sciences, Universidad Austral de Chile, Valdivia 511-0566, Chile; claudiaquezada@uach.cl (C.Q.); benjamin.guerrero@alumnos.uach.cl (B.G.); pamela.silva01@uach.cl (P.S.); 6 Millennium Institute on Immunology and Immunotherapy, Universidad Austral de Chile, Valdivia 511-0566, Chile; 7 Escuela de Medicina, Facultad de Medicina, Campus Tres Pascualas, Universidad San Sebastián, Lientur 1457, Concepción 407-0129, Chile; sebastian.alarcon@uss.cl; 8 Institute of Biomedical Science (ICB), Faculty of Medicine, Universidad Andrés Bello, Santiago 837-0183, Chile; vpolaveliz@gmail.com

**Keywords:** adenosine signaling, A2B adenosine receptor (A2BAR), diabetes mellitus (DM), diabetic lung disease (DLD), macrophage, macrophage polarization (M1/M2 balance), MRS1754, pulmonary fibrosis, pulmonary inflammation

## Abstract

The pathophysiological mechanisms underlying diabetic lung disease (DLD) remain poorly understood. Beyond hyperglycemia, dysregulated molecules such as adenosine may contribute to pulmonary inflammation and fibrosis. MRS1754, a selective antagonist of the A2B adenosine receptor (A2BAR), has demonstrated anti-inflammatory and antifibrotic effects, including reductions in macrophage (MΦ) infiltration in animal models of diabetes-associated complications; however, its effects on DLD remain unexplored. Here, diabetic Sprague-Dawley rats were treated with MRS1754, and pulmonary inflammation, fibrosis, MΦ infiltration, and polarization were assessed in bronchoalveolar lavage fluid (BALF) and lung tissue using immunohistopathological and molecular approaches. According to data normality, one-way ANOVA/Tukey’s test or Kruskal–Wallis/Dunn’s test were employed, as appropriate; ordinal data were analyzed using the Mann–Whitney test with Bonferroni correction. Compared with controls, diabetic rats exhibited pulmonary inflammation and fibrosis, increased MΦ infiltration, and an M1/M2 polarization imbalance. MRS1754 attenuated these alterations, reducing Tumor necrosis factor-alpha (TNF-α) and Transforming growth factor-beta 1 (TGF-β1) levels, decreasing MΦ accumulation in BALF and lung tissue, and fully restoring the M1/M2 balance to control levels. BALF MΦ populations, particularly the M2 (Cluster of differentiation 163-positive; CD163^+^) subset, correlated strongly with TGF-β1 and fibronectin (FN1) levels. Reanalysis of human fibrotic lung single-cell transcriptomic data further identified MΦ as a major TGF-β1-expressing cell population. Collectively, these findings suggest that A2BAR antagonism partially mitigates inflammation, fibrosis, and MΦ infiltration while fully restoring MΦ polarization balance in DLD, supporting its potential as a therapeutic strategy.

## 1. Introduction

Diabetes mellitus (DM) is a chronic metabolic disorder characterized by persistent hyperglycemia resulting from defects in insulin secretion and/or action [1,2,3]. It is strongly associated with modifiable risk factors such as sedentary lifestyle, obesity, smoking, and aging [1,2,3,4]. The global prevalence of diabetes has doubled over recent decades, increasing from approximately 7% in 1990 to 14% in 2022, affecting hundreds of millions of individuals worldwide [5]. According to the 2025 release of the 11th edition of the International Diabetes Federation (IDF) Diabetes Atlas, 589 million adults were living with diabetes worldwide in 2024, with projections reaching 853 million by 2050 [1]. This escalating prevalence is accompanied by a substantial economic burden [6], with global healthcare expenditures exceeding USD 1 trillion in 2024 [1]. These data underscore the urgent need for improved prevention strategies and more effective therapeutic approaches.

Chronic hyperglycemia is a major driver of vascular complications in DM, including microvascular (nephropathy, retinopathy, neuropathy) and macrovascular (coronary artery disease, cerebrovascular disease) damage [1,3,7]. Beyond these classical complications, emerging evidence identifies the lung as an additional target organ affected by diabetes [8,9,10,11]. Structural and functional abnormalities have been described, including alveolar epithelial injury, basement membrane thickening, reduced lung elasticity, and impaired gas exchange [8,9,10,12,13,14]. Histopathological analyses in both human and experimental models demonstrate septal fibrosis, extracellular matrix (ECM) deposition, and alveolar remodeling [12,14,15]. Collectively, these findings support the recognition of diabetic lung disease (DLD) as a distinct diabetes-associated complication [11,16]. However, DLD remains underrecognized in both research frameworks and clinical practice.

Immune dysfunction represents another hallmark of DM. Sustained hyperglycemia alters the number, polarization (M1 or M2 phenotype), and functional activity of immune cells, particularly macrophages (MΦ) [17,18,19,20,21,22]. Experimental evidence indicates that chronic hyperglycemia reduces MΦ counts and disrupts their polarization balance and effector functions [23,24,25,26]. This dysregulation contributes to the increased susceptibility to infections observed in diabetic patients [27]. Furthermore, enhanced polarization toward a pro-fibrotic M2 MΦ phenotype has been reported in diabetic models and in bone marrow-derived MΦ exposed to high-glucose conditions [17,26,27], potentially accelerating tissue fibrosis. During normal tissue repair, MΦ regulate ECM remodeling and clearance of apoptotic cells; however, these processes are frequently impaired in diabetes [28,29].

In addition to hyperglycemia, extracellular stress mediators may contribute to DLD pathophysiology. Adenosine, an immunomodulatory purine nucleoside released during metabolic stress and tissue injury, has emerged as a critical regulator of inflammation and fibrosis [30,31,32,33]. Under conditions of cellular stress, hypoxia, or inflammation, adenosine is primarily generated from the extracellular breakdown of adenosine triphosphate (ATP), which is actively released by damaged, stressed, or dying cells. Extracellular ATP is sequentially hydrolyzed by ectonucleotidases such as Cluster of differentiation 39 (CD39) and Cluster of differentiation 73 CD73, leading to the accumulation of extracellular adenosine [33]. Therefore, sustained metabolic imbalance and tissue injury, as observed in diabetes, may promote increased ATP release and subsequent adenosine production within the pulmonary microenvironment.

Adenosine signals through four G protein-coupled receptors (A1, A2A, A2B, and A3), exerting context-dependent effects on immune and structural cells [31,32,33,34,35,36,37]. Among these receptors, A2B (A2BAR) is of particular interest due to its low affinity for adenosine and preferential activation under conditions of elevated extracellular adenosine, such as chronic inflammation and diabetes [31,32,35,37]. A2BAR signaling promotes cytokine production, fibroblast activation, and collagen synthesis, thereby contributing to pathological tissue remodeling [31,36,37]. Its involvement has been demonstrated in renal, hepatic, and cardiac diabetic complications [30,31,32,33,38,39,40], and A2BAR antagonism has shown therapeutic potential in multiple models of metabolic and fibrotic disease [37,41,42,43].

Adenosine also modulates MΦ activation and differentiation [36,37,44,45]. Activation of A2BAR enhances the development of pro-fibrotic M2 MΦ phenotypes, amplifying tissue remodeling responses. In pulmonary contexts, A2BAR mediates pro-fibrotic signaling in lung fibroblasts and promotes airway remodeling [41,42]. Genetic deletion of A2BAR attenuates chronic pulmonary inflammation [46]; however, its specific role in diabetic lung injury remains unclear. MRS1754, a selective A2BAR antagonist, has demonstrated anti-inflammatory and antifibrotic effects in diabetic nephropathy models, including reductions in MΦ infiltration and myofibroblastic differentiation [35,36,37,40]. Pharmacological blockade of A2BAR also decreases Transforming growth factor-beta 1 (TGF-β1) expression and attenuates glomerular fibrosis in diabetic rats [47]. Nevertheless, whether A2BAR antagonism mitigates inflammation, fibrosis, and MΦ infiltration and polarization in DLD remains unknown.

In the present study, we investigated whether A2BAR antagonism alleviates lung injury in DLD. We hypothesized that pharmacological blockade of A2BAR with MRS1754 attenuates pulmonary inflammation and fibrosis and modulates MΦ infiltration and polarization in a rat model of DLD, thereby supporting A2BAR as a potential therapeutic target.

## 2. Results

### 2.1. In Vivo Blockade of A2BAR with MRS1754 Modulates the Extracellular Adenosine Axis and A2BAR Expression in the Lungs of Diabetic Rats

**Figure 1:**
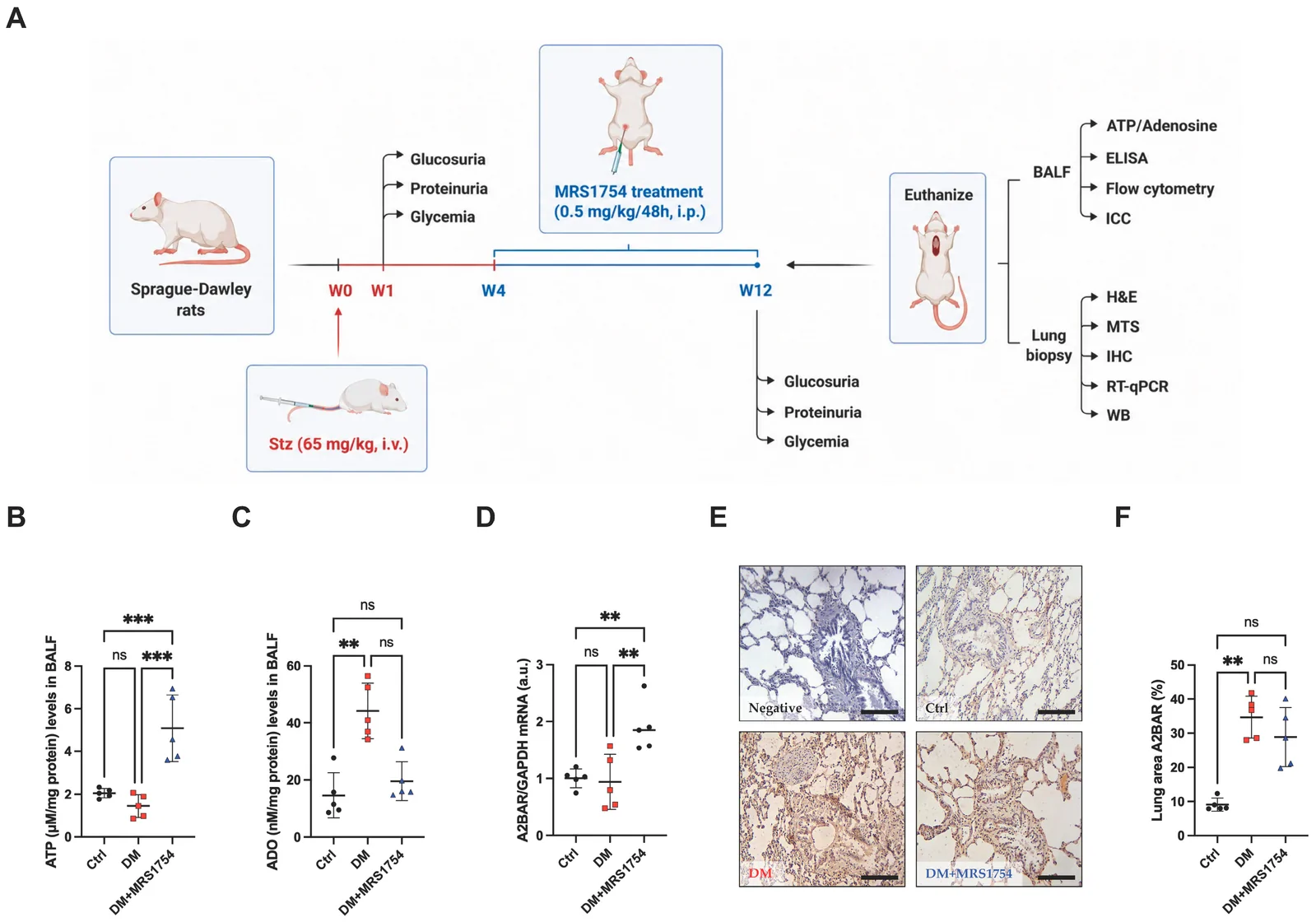
Experimental timeline and changes in ATP and adenosine levels in BALF, and A2BAR mRNA and protein expression in lung tissue of diabetic rats treated with MRS1754. (**A**) Experimental timeline. Male Sprague-Dawley rats (6 weeks old; 200–250 g) received a single intravenous (i.v.) injection of streptozotocin (STZ; 65 mg/kg) at week 0 (W0) to induce diabetes mellitus. One week later (W1), body weight, blood glucose, proteinuria, and glucosuria were assessed. Four weeks after STZ administration (W4), diabetic rats began intraperitoneal (i.p.) treatment with the A2B adenosine receptor (A2BAR) antagonist MRS1754 (0.5 mg/kg every 48 h) for eight weeks. At week 12 (W12), animals were euthanized, and bronchoalveolar lavage fluid (BALF) and lung tissue were collected for biochemical, molecular, and histological analyses. (**B**) Adenosine triphosphate (ATP) and (**C**) adenosine (ADO) concentrations in BALF normalized to protein concentration and expressed per mg of protein. (**D**) A2BAR transcript levels quantified by reverse transcription quantitative polymerase chain reaction (RT-qPCR) and expressed as relative expression using the delta-delta cycle threshold (2^−ΔΔCt^) method, with glyceraldehyde-3-phosphate dehydrogenase (*Gapdh*) as the reference gene. (**E**) Representative immunohistochemical staining for A2BAR in lung sections from control non-diabetic (Ctrl), diabetic (DM), and diabetic MRS1754-treated (DM+MRS1754) groups. The negative control corresponds to sections processed without primary antibody. Magnification: 100× (scale bar = 200 µm) (**F**) Quantitative analysis of the percentage of A2BAR-positive area in whole lung tissue. Seven non-overlapping fields per animal were analyzed and averaged to obtain a single value per animal. Graphs are presented as mean ± standard deviation (SD) and each dot represents the mean value obtained from one animal (*n* = 5 animals per group). Statistical analysis for panel (**B**,**D**) was performed using one-way analysis of variance (ANOVA) followed by Tukey’s multiple comparison test. Statistical analysis for panels (**C**,**F**) was performed using the Kruskal–Wallis test followed by Dunn’s multiple comparison test. Statistical significance is indicated as ** *p* < 0.01, *** *p* < 0.001; ns, not significant. Abbreviations: W0, week zero; W1, week one; W4, week four; W12, week twelve; STZ, streptozotocin; i.v., intravenous; i.p., intraperitoneal; BALF, bronchoalveolar lavage fluid; ADO, adenosine; ATP, adenosine triphosphate; A2B adenosine receptor (A2BAR); ELISA, enzyme-linked immunosorbent assay; H&E, Hematoxylin and Eosin; MTS, Masson’s trichrome staining; IHC, immunohistochemistry; ICC, immunocytochemistry; RT-qPCR, reverse transcription quantitative polymerase chain reaction; and WB, Western blotting.

The experimental timeline is shown in Figure 1A. To determine whether adenosine signaling is altered in DLD, we quantified key components of the extracellular adenosine axis, including ATP levels as a major precursor of adenosine [34], and adenosine concentrations in bronchoalveolar lavage fluid (BALF). Additionally, A2BAR mRNA and protein expression were measured in lung tissue from control non-diabetic (Ctrl), diabetic (DM), and DM+MRS1754 rats.

ATP levels did not differ between Ctrl (2.05 µM/mg protein) and DM (1.45 µM/mg protein) animals (*p* = 0.5974); however, MRS1754 treatment significantly increased ATP levels (5.08 µM/mg protein) by ~2.5- and ~3.5-fold compared with the Ctrl (*p* = 0.0008) and DM (*p* = 0.0002) groups, respectively (Figure 1B).

In contrast, adenosine levels were ~3.0-fold higher in DM rats (44.22 nM/mg protein) than in Ctrl animals (14.63 nM/mg protein) (*p* = 0.0044). Although MRS1754-treated rats showed a tendency toward lower adenosine levels (19.57 nM/mg protein), this reduction did not reach statistical significance compared with the DM group (*p* = 0.1017), and no significant difference was observed between DM+MRS1754 and Ctrl animals (*p* = 0.8665) (Figure 1C).

A2BAR mRNA (*Adora2b*) levels in lung tissue did not differ significantly between DM and Ctrl rats (*p* = 0.9654). MRS1754 treatment significantly increased A2BAR transcript expression in DM+MRS1754 rats compared with both Ctrl (~1.9-fold, *p* = 0.0089) and DM groups (~2.0-fold, *p* = 0.0057) (Figure 1D). To further support this finding at the transcriptional level, we reanalyzed an independent, diabetes-specific rat lung microarray dataset (GEO accession GSE15900) [48], comprising whole-lung gene expression profiles from non-diabetic control (*n* = 5) and streptozotocin (STZ)-induced diabetic (*n* = 7) Wistar rats. Consistent with our reverse transcription quantitative polymerase chain reaction (RT-qPCR) results, *Adora2b* transcript levels did not differ significantly between diabetic and control lungs in this independent cohort (*p* = 0.432, Mann–Whitney U; Figure S1A), reinforcing that A2BAR is not transcriptionally upregulated in the diabetic lung. A broader screen of 22 additional adenosine axis transcripts (receptors, ectonucleotidases, nucleoside transporters, and adenosine-metabolizing enzymes) in this same dataset is provided in Table S1 for reference.

To assess which pulmonary cell populations express *ADORA2B* in the human lung, we reanalyzed publicly available single-cell RNA-sequencing data from control and fibrotic lung tissue (GEO GSE121611/GSE122960) [49]. *ADORA2B* was broadly but sparsely expressed across the dataset, detected in 3.3% of all cells, with similar detection rates in MΦ (3.5%) and epithelial cells (3.6%) (Figure S1B). Notably, *ADORA2B* expression was consistently detected within aberrant basaloid-like epithelial populations—a disease-restricted epithelial state described in fibrotic interstitial lung disease (ILD) across four independent human datasets [49,50,51,52] and absent from healthy control lung in each of these studies (Figure S1C). In an additional, not yet peer-reviewed dataset comprising monocyte/MΦ subpopulations from control, chronic obstructive pulmonary disease (COPD) and idiopathic pulmonary fibrosis (IPF) lungs [53], *ADORA2B* expression trended higher in IPF relative to control across most monocyte/MΦ subtypes, though this comparison should be interpreted with caution given its preprint status (Figure S1D).

At the protein level, A2BAR immunoreactivity, expressed as the percentage of positive area, was significantly increased in the DM group (34.86%) compared with Ctrl animals (8.4%) (*p* = 0.0089). Although DM+MRS1754 rats showed an elevated percentage of A2BAR-positive area (28.62%) relative to Ctrl, this difference did not reach statistical significance (*p* = 0.0589). No significant difference in the percentage of A2BAR-positive area was observed between the DM and DM+MRS1754 groups (*p* > 0.9999), indicating that MRS1754 treatment did not reduce A2BAR protein expression at the tissue level (Figure 1E,F). To indirectly assess A2BAR activation in the lung, we evaluated three downstream signaling branches associated with A2BAR signaling: (i) Nuclear factor kappa B (NF-κB) activation (phospho-Ser536/total NF-κB ratio), (ii) SMAD family member 3 (SMAD3) phosphorylation (phospho-Ser423/total SMAD3 ratio), and (iii) Phosphoinositide 3-kinase (PI3K)/Protein kinase B (PKB or Akt) pathway activation (phospho-Ser473/total Akt1 ratio) [40]. Densitometric analysis of these three downstream targets did not reveal a consistent pattern across experimental groups, and no statistically significant differences were observed between Ctrl, DM, and DM+MRS1754 for any of the three phosphorylation/total protein ratios (Figure S2).

Collectively, these findings suggest that the extracellular adenosine axis is activated in DLD, as evidenced by elevated adenosine levels and increased A2BAR protein expression in diabetic rats, without a concomitant increase in A2BAR mRNA—a pattern independently corroborated in an unrelated diabetic rat lung microarray dataset. Although MRS1754 treatment did not significantly reduce adenosine concentrations or A2BAR protein levels, the marked increase in ATP levels and A2BAR transcript expression following A2BAR blockade suggests a functional shift in extracellular nucleotide metabolism and a compensatory transcriptional response. Densitometric analysis of three downstream signaling branches associated with A2BAR signaling (NF-κB, SMAD3, and Akt phosphorylation) did not reveal a consistent or statistically significant pattern across experimental groups, indicating that the functional consequences of A2BAR engagement in this model are not readily captured by these canonical signaling readouts alone. Nevertheless, single-cell transcriptomic data from human fibrotic lung indicate that *ADORA2B* is consistently expressed within aberrant basaloid-like epithelial populations and MΦ across multiple independent cohorts, supporting the translational relevance of A2BAR as a receptor engaged in fibrotic lung remodeling. Together, these findings indicate that A2BAR is engaged at the protein level in diabetic lung, and that its pharmacological blockade produces measurable downstream effects on the adenosine axis, even though the precise intracellular signaling mechanism linking A2BAR to the inflammatory and fibrotic phenotype remains to be fully defined.

### 2.2. A2BAR Antagonism Reduces Histopathological and Cytokine Markers of Pulmonary Inflammation in Diabetic Rats

**Figure 2:**
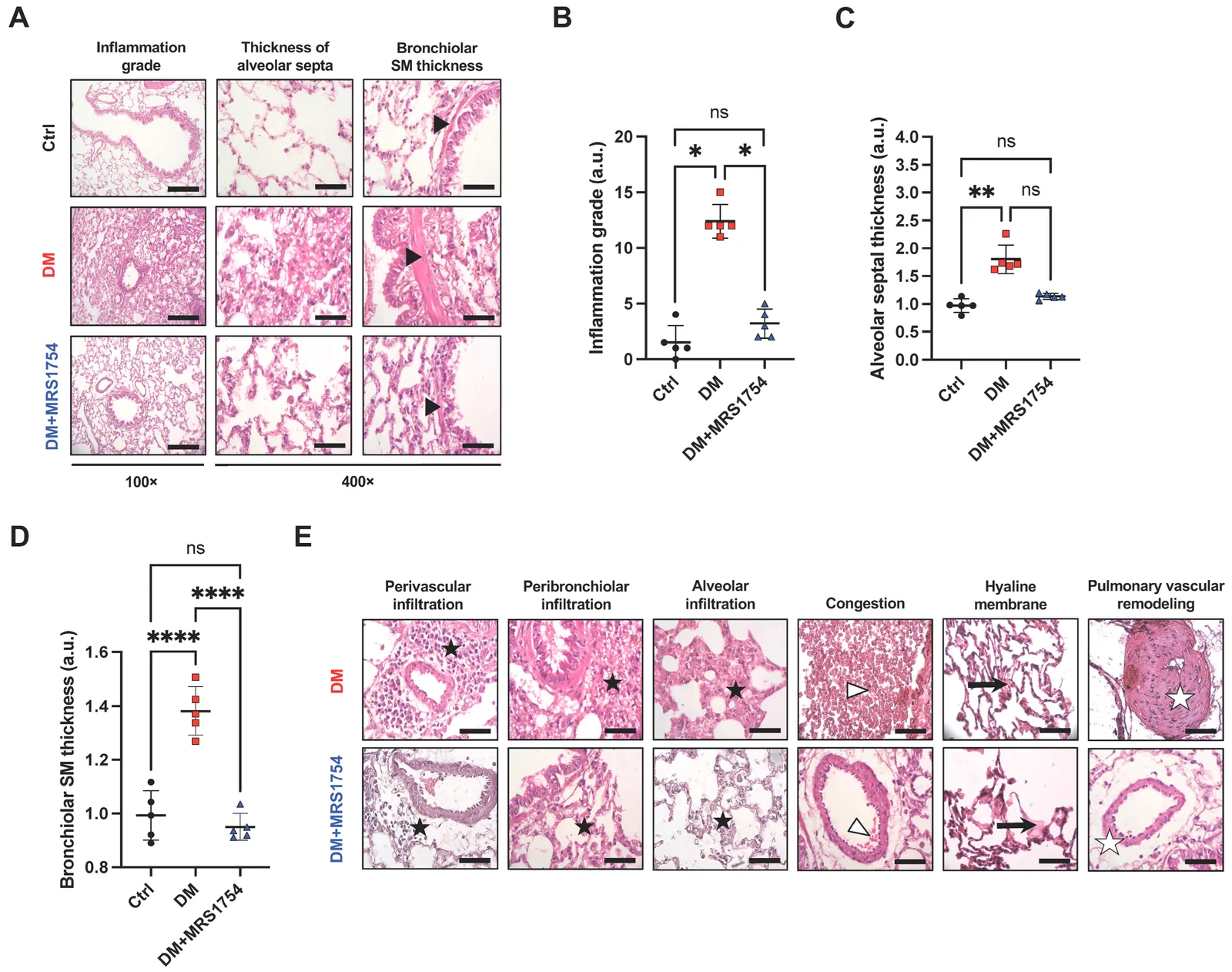
Effect of in vivo MRS1754 treatment on the degree of histopathological lung inflammation in diabetic rats. (**A**) Representative H&E-stained lung sections from control non-diabetic (Ctrl), diabetic (DM), and diabetic MRS1754-treated (DM+MRS1754) rats. Low-magnification images (100×; scale bar = 200 µm) illustrate the overall degree of pulmonary inflammation. Higher magnification (400×; scale bar = 50 µm) highlights regions of interest showing alveolar septal thickness and bronchiolar smooth muscle (SM) thickness. Black arrowheads (►) indicate bronchiolar SM. (**B**–**D**) Quantitative analysis showing individual data points and median ± interquartile range (IQR) for (**B**) inflammation grade and mean ± SD for (**C**) alveolar septal thickness, and (**D**) bronchiolar SM thickness in Ctrl, DM, and DM+MRS1754 groups. Data in (**C**,**D**) are expressed in arbitrary units (a.u.) and were calibrated to the control group (set to 1). (**E**) Representative images (400×; scale bar = 50 µm) showing perivascular, peribronchiolar, and alveolar inflammatory cell infiltration, vascular congestion, hyaline membrane formation, and pulmonary vascular remodeling in DM and DM+MRS1754 lungs. Black stars (★) indicate inflammatory cell infiltration, white arrowheads (▷) indicate vascular congestion (erythrocytes), arrows (→) indicate hyaline membrane formation, and white stars (☆) indicate pulmonary vascular remodeling. For graphs (**C**,**D**), seven non-overlapping fields per animal were analyzed and averaged to obtain a single value per animal. Each dot represents one animal (*n* = 5 animals per group). Statistical analysis for panel (**B**) was performed using the Mann–Whitney test with Bonferroni correction for multiple comparisons. Statistical analysis for panel (**C**) was performed using the Kruskal–Wallis test followed by Dunn’s multiple comparison test. Statistical analysis for panel (**D**) was performed using one-way ANOVA followed by Tukey’s multiple comparison test. Statistical significance is indicated as * *p* < 0.05, ** *p* < 0.01, **** *p* < 0.0001; ns, not significant.

Given that inflammatory processes have been described in DLD [15,16], and that A2BAR is a recognized modulator of inflammation [30,43], we assessed the pulmonary anti-inflammatory effects of in vivo A2BAR antagonism by treating diabetic rats with MRS1754 (0.5 mg/kg every 48 h) for 8 weeks (Figure 1A). At the end of this period, rats were euthanized, and lung biopsies were taken to assess the degree of inflammation using a histopathological damage score (Table 1). This analysis showed that the lungs of Ctrl rats had a normal morphology without inflammation (grade 2/15), while the lungs of DM rats presented severe inflammation (grade 12/15) (*p* = 0.0237 vs. Ctrl) that was attenuated by treatment with MRS1754 (grade 3/15) (*p* = 0.0237 vs. DM), restoring inflammation to levels comparable to those of the Ctrl group (*p* = 0.2382) (Table 1 and Figure 2A,B and Figure S3).

In contrast to the Ctrl group, DM rats demonstrated a ~80% increase in alveolar septal thickness (*p* = 0.0027) with an evident decrease in alveolar air spaces, and bronchiolar SM thickness was ~39% greater than in the Ctrl group (*p* < 0.0001) (Figure 2A,C,D and Figure S3). Additionally, perivascular, peribronchiolar and alveolar inflammatory cell infiltration, vascular congestion, hyaline membrane formation, and pulmonary vascular remodeling were observed in the lung of DM rats (Table 1 and Figure 2E).

Although MRS1754 treatment did not significantly reduce alveolar septal thickness compared with the DM group (*p* = 0.1431), a tendency toward decreased thickness was observed (~37% reduction), accompanied by a concomitant recovery of alveolar air space (Figure 2A,C and Figure S3). In contrast, bronchiolar SM thickness was significantly reduced by ~31% in DM+MRS1754 rats relative to the DM group (*p* < 0.0001), restoring values comparable to those of the Ctrl group (*p* = 0.6855) (Figure 2A,D and Figure S3). Similarly, perivascular, peribronchiolar and alveolar inflammatory cell infiltration, vascular congestion, hyaline membrane formation, and pulmonary vascular remodeling were all attenuated in DM+MRS1754 rats compared with the DM group (Table 1 and Figure 2E).

Interleukin-1β (IL-1β), Interleukin-6 (IL-6), and Tumor Necrosis Factor-α (TNF-α) are key pro-inflammatory cytokines associated with multiple inflammatory lung diseases and metabolic disorders, including diabetes [54,55]. However, their specific contribution to DLD remains insufficiently characterized [11]. IL-1β expression was assessed exclusively at the protein level by immunohistochemistry (IHC), while *Il6* (encoding IL-6) mRNA and TNF-α protein levels were quantified in lung tissue and BALF, respectively.

**Figure 3:**
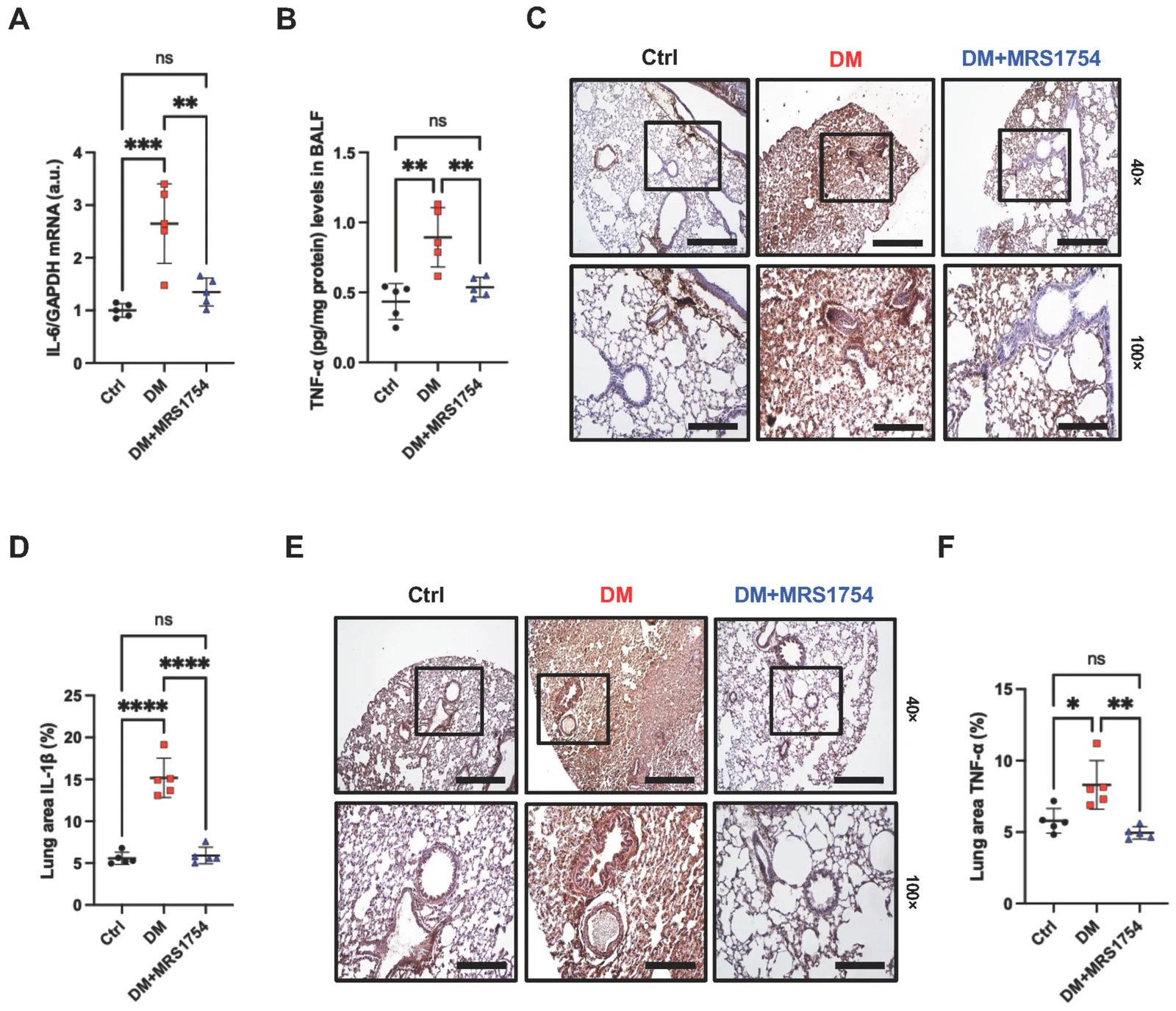
Effects of in vivo MRS1754 treatment on inflammatory marker expression in BALF and lung tissue of diabetic rats. Interleukin-6 (IL-6) (**A**) mRNA expression in lung tissue and Tumor Necrosis Factor-α (TNF-α) (**B**) protein concentration in BALF were evaluated in Ctrl, DM, and DM+MRS1754 rats by RT-qPCR (**A**) and ELISA (**B**), respectively. RT-qPCR data were normalized to *Gapdh* and were calibrated to the control group (set to 1). ELISA results were normalized to protein concentration and expressed per mg of protein in BALF. Lung sections from Ctrl, DM, and DM+MRS1754 rats were immunostained with anti-Interleukin-1β (IL-1β) (**C**) and anti-TNF-α (**E**) antibodies. Representative images show lung sections (40× magnification; scale bar = 500 µm) and a higher-magnification view (100× magnification; scale bar = 200 µm) of a selected region (solid-line square) containing bronchioles, blood vessels, and alveolar septa. Quantitative analysis shows the percentage of IL-1β (**D**) and TNF-α (**F**) immunoreactivity in whole lung tissue. In graphs (**A**,**B**), each dot represents one animal (*n* = 5 animals per group). For graphs (**D**,**F**), seven non-overlapping fields per animal were analyzed and averaged to obtain a single value per animal. Each dot represents one animal (*n* = 5 animals per group). Graphs are presented as mean ± SD. Statistical analysis for all panels was performed using one-way ANOVA followed by Tukey’s multiple comparison test. Statistical significance is indicated as * *p* < 0.05, ** *p* < 0.01, *** *p* < 0.001, and **** *p* < 0.0001; ns, not significant.

The mRNA expression level of *Il6* in lung tissue increased ~2.64-fold in the DM group compared with the Ctrl group (*p* = 0.0003), but it was reduced by ~49% following treatment with MRS1754 (*p* = 0.0023), restoring values to levels comparable to those of the Ctrl group (*p* = 0.4842) (Figure 3A). Additionally, TNF-α levels in the BALF of DM (0.89 pg/mg protein) rats increased ~2.06-fold compared with the Ctrl group (0.43 pg/mg protein) (*p* = 0.0010) and were reduced by ~40% following MRS1754 treatment (0.53 pg/mg protein) (*p* = 0.0068), restoring values to levels comparable to those of the Ctrl group (*p* = 0.5384) (Figure 3B).

To extend these findings and define their tissue-level distribution, we examined the compartment-specific localization of these cytokines within the lung (bronchiolar, vascular, and alveolar regions) by IHC. The percentage of IL-1β immunostaining was significantly increased in the lungs of DM rats (15.19%) compared with Ctrl animals (5.572%), representing an approximately 2.73-fold elevation (*p* < 0.0001) (Figure 3C,D). MRS1754 treatment markedly reduced IL-1β-positive staining to 5.913%, corresponding to an approximately 2.57-fold decrease (~61% reduction) relative to the DM group (*p* < 0.0001), restoring values to levels comparable to those of the Ctrl group (*p* = 0.9339) (Figure 3C,D).

Compartment-specific analysis revealed that IL-1β immunostaining was markedly elevated in bronchiolar (5.55-fold), vascular (2.80-fold), and alveolar (2.08-fold) regions of DM rats compared with Ctrl animals (Figure S4). MRS1754 treatment significantly reduced IL-1β-positive area in the vascular (~66% reduction, *p* = 0.0327) and alveolar (~56% reduction, *p* < 0.0001) compartments relative to DM rats, restoring values to levels comparable to those of the Ctrl group (Figure S4). In the bronchiolar compartment, a tendency toward reduced IL-1β staining was observed (~70% reduction) following MRS1754 treatment; however, this did not reach statistical significance (*p* = 0.1017) (Figure S4).

Similarly, TNF-α-positive staining was significantly increased in the lungs of DM rats (8.308%) compared with Ctrl animals (5.792%), representing an approximately 1.43-fold elevation (*p* = 0.0108) (Figure 3E,F). In vivo A2BAR antagonism reduced TNF-α staining to 4.950%, corresponding to an approximately 40% decrease relative to DM rats (*p* = 0.0014), restoring values to levels comparable to those in Ctrl group (*p* = 0.4874) (Figure 3E,F). Compartment-specific analysis demonstrated increased TNF-α immunostaining in bronchiolar (1.67-fold, *p* = 0.0005) and alveolar (~1.73-fold, *p* < 0.0001) regions of DM rats compared with Ctrl animals (Figure S5). Treatment with MRS1754 significantly reduced TNF-α-positive area by ~58% in the bronchiolar compartment (*p* < 0.0001) and by ~45% in the alveolar compartment (*p* < 0.0001) relative to DM rats, restoring values to levels comparable to those of the Ctrl group in both cases (Figure S5). No significant differences were observed in TNF-α immunostaining within the vascular compartment among groups (*p* > 0.05) (Figure S5).

Collectively, these findings suggest that in vivo A2BAR antagonism with MRS1754 partially attenuates the histopathological, structural, and molecular hallmarks of pulmonary inflammation in diabetic rats, with effects that are compartment-specific and not uniform across all inflammatory markers, supporting a modulatory rather than a global anti-inflammatory role for A2BAR signaling in DLD.

### 2.3. A2BAR Antagonism Attenuates Compartment-Specific Fibrotic Remodeling in the Lungs of Diabetic Rats

**Figure 4:**
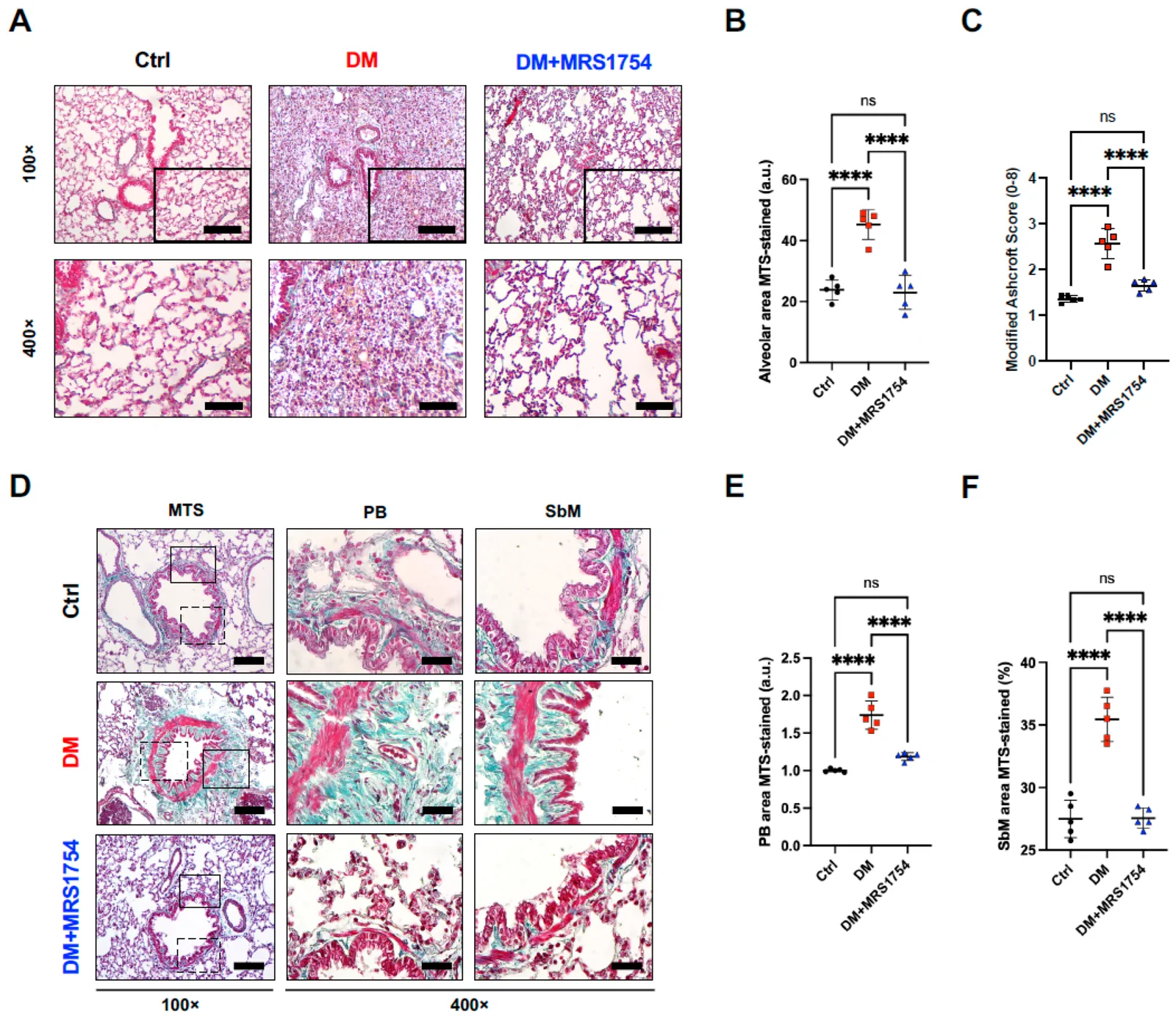
Effect of in vivo A2BAR antagonism on pulmonary ECM deposition in diabetic rats. (**A**) Representative images of Masson’s trichrome staining (MTS) showing overall ECM distribution in whole lung sections (100× magnification, scale bar = 400 µm) and a higher-magnification view of the alveolar region (400×; solid-line inset; scale bar = 100 µm) from Ctrl, DM, and DM+MRS1754 rats. (**B**) Quantification of MTS-stained alveolar area (%) in Ctrl, DM, and DM+MRS1754 rats. (**C**) Quantification of modified Ashcroft score in Ctrl, DM, and DM+MRS1754 rats, assessed on hematoxylin–eosin- and Masson’s trichrome-stained sections. (**D**) Representative MTS images showing ECM distribution in the bronchiolar region at low (100×, scale bar = 200 µm) and high (400×; scale bar = 50 µm) magnification. Insets highlight the peribronchiolar (PB; solid-line square) and submucosal (SbM; dashed-line square) areas of the bronchioles. (**E**) Quantification of MTS-stained PB area (arbitrary units, a.u.) and (**F**) MTS-stained SbM area (%) in Ctrl, DM, and DM+MRS1754 rats. For graphs (**B**,**E**,**F**), seven non-overlapping fields per animal were analyzed and averaged to obtain a single value per animal. For graph (**C**), 25 non-overlapping fields per animal (hematoxylin–eosin- and Masson’s trichrome-stained sections) were analyzed and averaged to obtain a single value per animal. Each dot represents one animal (*n* = 5 animals per group). Data in (**E**) were normalized to the control group (set to 1). Graphs are presented as mean ± SD. Statistical analysis for all panels was performed using one-way ANOVA followed by Tukey’s multiple comparison test. Statistical significance is indicated as **** *p* < 0.0001; ns, not significant.

In recent years, several studies have successfully validated the lung as a target organ for fibrosis during diabetes [10,11,14,15,16,56]. To assess pulmonary fibrosis, Masson’s trichrome staining (MTS) was used to detect ECM accumulation, in particular type I collagen fibers [57,58]. MTS staining revealed increased ECM deposition across multiple pulmonary compartments in DM rats compared with Ctrl animals (Figure 4 and Figure S6). Alveolar ECM content was ~1.90-fold higher in DM rats (45.20%) than in Ctrl animals (23.80%) (*p* < 0.0001) and was reduced by ~49% (22.99%) following MRS1754 treatment (*p* < 0.0001), with values returning to control levels (*p* = 0.9596) (Figure 4A,B). Consistent with these findings, semiquantitative histopathological assessment using the modified Ashcroft score corroborated the increased fibrotic remodeling in DM rats. The modified Ashcroft score was ~1.88-fold higher in DM rats (2.57) than in Ctrl animals (1.37) (*p* < 0.0001) and was reduced by ~36% (1.66) following MRS1754 treatment (*p* < 0.0001), with values not significantly different from control levels (*p* = 0.1067) (Figure 4C). A similar pattern was observed in the peribronchiolar (PB) and submucosal (SbM) areas of the bronchioles, where ECM content was ~1.74-fold and ~1.29-fold higher in DM rats than in Ctrl animals, respectively (*p* < 0.0001 for both). MRS1754 treatment significantly reduced PB ECM by ~32% (*p* < 0.0001) and SbM ECM by ~22% (*p* < 0.0001), restoring both to control levels (*p* = 0.0554 and *p* = 0.9983, respectively) (Figure 4D–F).

Collectively, these findings—corroborated by semiquantitative histopathological scoring—suggest that in vivo A2BAR antagonism with MRS1754 mitigates pulmonary fibrosis in diabetic rats by reducing ECM and type I collagen fiber accumulation across the alveolar, peribronchiolar, and submucosal compartments of the lung.

In fibrotic lung remodeling, fibronectin (FN), α-smooth muscle actin (α-SMA), TGF-β1, and the type I collagen α1 chain (COL1A1) are widely used markers of myofibroblast activation and ECM accumulation; these molecules are consistently elevated in fibrotic processes, including lung fibrosis [51,59,60,61,62,63,64]. In diabetes-related lung injury, preclinical work points to a hyperglycemia–TGF-β1 axis that increases FN, α-SMA, and collagen deposition, although human evidence remains limited [65,66]. Guided by this framework, we quantified *Fn1* (encoding FN), *Acta2* (encoding α-SMA), and *Tgfb1* (encoding TGF-β1) mRNA expression by RT-qPCR, TGF-β1 protein levels in BALF by ELISA, and COL1α1 and TGF-β1 protein distribution in lung tissue by IHC, as primary indicators of profibrotic activation.

**Figure 5:**
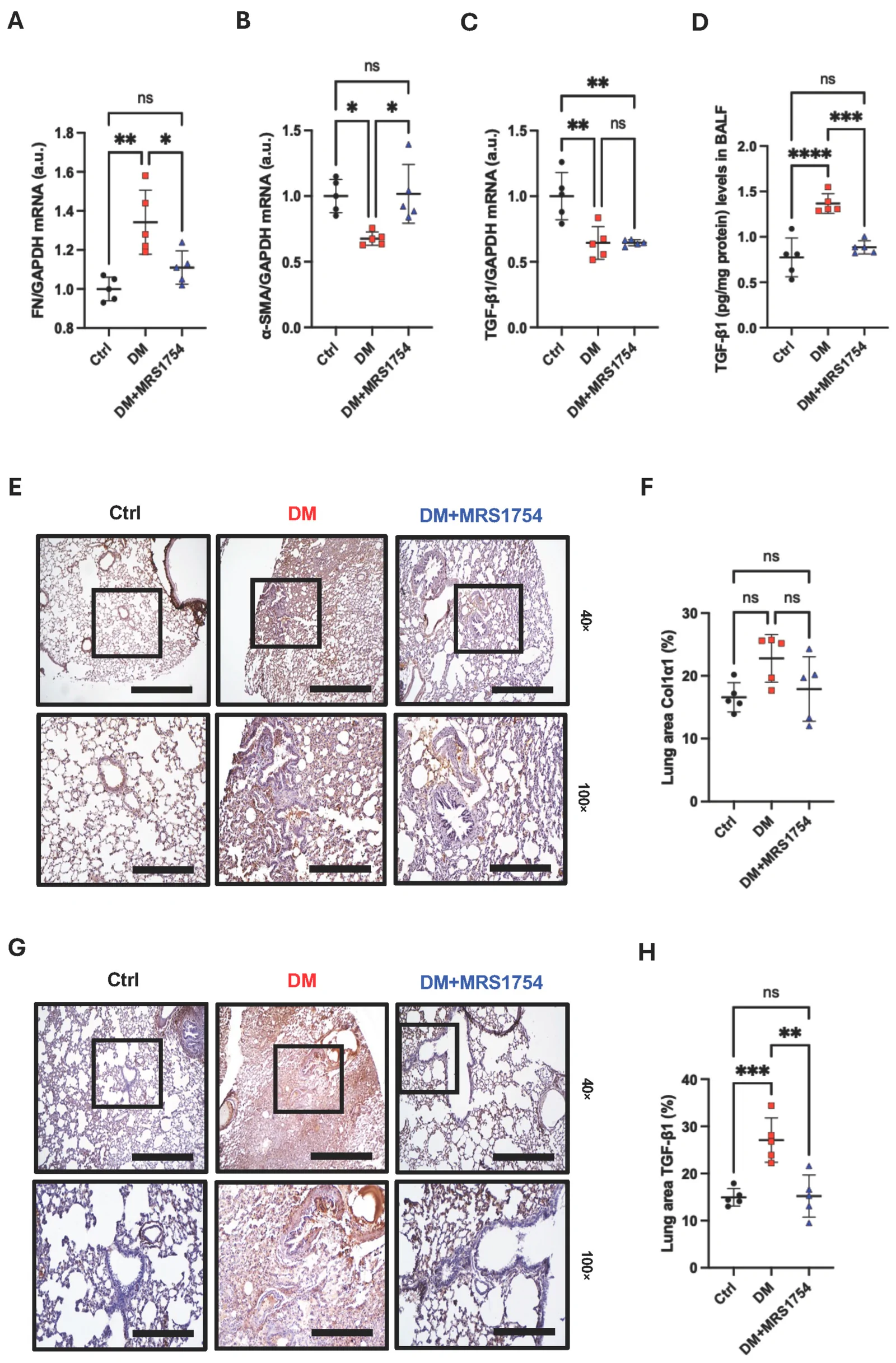
Effect of in vivo MRS1754 treatment on the expression of fibrotic markers in the BALF and lung tissue of diabetic rats. The expression of *Fn1* (**A**), *Acta2* (**B**) and *Tgfb1* (**C**) was evaluated by RT-qPCR in lung tissue of control Ctrl, DM, and DM+MRS1754 rats. RT-qPCR data were normalized to *Gapdh* and were calibrated to the control group (set to 1). Additionally, TGF-β1 concentration was measured by ELISA in the BALF (**D**). ELISA results were normalized to protein concentration and expressed per mg of protein in BALF. In graphs (**A**–**D**) each dot represents the mean value obtained per animal (*n* = 5 animals per group). Histological sections of lungs from Ctrl, DM, and DM+MRS1754 rats were immunostained with anti-COL1α1 (**E**) and anti-TGF-β1 (**G**) antibodies. Representative images show lung sections (40× magnification, scale bar = 625 µm) and specific compartments such as bronchioles, blood vessels, and alveolar septa (100× magnification, scale bar = 200 µm). Quantitative analysis shows the percentage of positive area for COL1α1 (**F**) and TGF-β1 (**H**) in whole lung tissue. For graphs (**F**,**H**), seven non-overlapping fields per animal were analyzed and averaged to obtain a single value per animal. Each dot represents one animal (*n* = 5 animals per group). Graphs are presented as mean ± SD. Statistical analysis was performed using one-way ANOVA followed by Tukey’s multiple comparison test. Statistical significance is indicated as * *p* < 0.05, ** *p* < 0.01, *** *p* < 0.001, and **** *p* < 0.0001; ns indicates no significant difference.

The mRNA expression of *Fn1* increased ~1.34-fold in DM rats compared with Ctrl (*p* = 0.0012) and was significantly reduced by ~17% following MRS1754 treatment (*p* = 0.0174), with values no longer significantly different from the Ctrl group (*p* = 0.3054) (Figure 5A). In contrast, *Acta2* and *Tgfb1* transcripts were significantly decreased in DM rats relative to Ctrl by ~33% (*p* = 0.0140) and ~36% (*p* = 0.0021), respectively (corresponding to ~0.68-fold and ~0.64-fold change, respectively; Figure 5B,C). Notably, in vivo A2BAR antagonism significantly restored *Acta2* mRNA expression in DM+MRS1754 animals relative to the DM group (~1.51-fold increase, *p* = 0.0102), reaching levels statistically indistinguishable from Ctrl rats (*p* = 0.9831; Figure 5B). In contrast, *Tgfb1* mRNA levels remained unchanged after MRS1754 treatment relative to the DM group (*p* = 0.9996) and continued to differ significantly from Ctrl (*p* = 0.0022; Figure 5C), indicating that A2BAR antagonism did not reverse the diabetes-induced downregulation of TGF-β1 transcription.

TGF-β1 protein concentrations in BALF, assessed by ELISA, were significantly elevated in DM rats (1.367 pg/mg protein) compared with Ctrl (0.7739 pg/mg protein; ~1.77-fold increase, *p* < 0.0001). This increase was significantly attenuated in the DM+MRS1754 group (0.8856 pg/mg protein), representing a ~35.3% reduction relative to untreated DM animals (*p* = 0.0005) and bringing values back in line with the Ctrl group (*p* = 0.4619) (Figure 5D).

IHC revealed that the mean percentage of total positive area for COL1α1 in the lungs of Ctrl, DM, and DM+MRS1754 groups was ~16.6%, ~23%, and ~18%, respectively, with no statistically significant differences among groups (Ctrl vs. DM, *p* = 0.0674; Ctrl vs. DM+MRS1754, *p* = 0.8576; DM vs. DM+MRS1754, *p* = 0.1635) (Figure 5E,F). When analyzed by compartment, bronchiolar regions showed values of ~9.7%, ~18%, and ~5% for Ctrl, DM, and DM+MRS1754, respectively. In this compartment, diabetes was associated with a nonsignificant tendency toward increased COL1α1 positivity (~87% increase; *p* = 0.1549 vs. Ctrl), whereas A2BAR antagonism significantly reduced positivity by ~72% relative to DM (*p* = 0.0049), abolishing the difference relative to the Ctrl group (*p* = 0.6867) (Figure S7). In the vascular compartment, mean percentages were ~27.8%, ~28.0%, and ~21.0% for Ctrl, DM, and DM+MRS1754, respectively, with no significant differences among groups (Ctrl vs. DM, *p* = 0.9989; Ctrl vs. DM+MRS1754, *p* = 0.2615; DM vs. DM+MRS1754, *p* = 0.2453) (Figure S7). In alveolar regions, values were ~11.4%, ~23.3%, and ~11.8% for Ctrl, DM, and DM+MRS1754, respectively. Diabetes significantly increased COL1α1 positivity by ~104% (~2.04-fold) compared with Ctrl animals (*p* = 0.0007), whereas MRS1754 treatment significantly reduced COL1α1 staining by ~49% relative to DM rats (*p* = 0.0009), normalizing values relative to the Ctrl group (*p* = 0.9838) (Figure S7).

Given that TGF-β1 is a key regulator of COL1α1 expression [61,62,63,64], we next evaluated its pulmonary distribution. The mean percentage of total positive area for TGF-β1 was ~14.96%, ~27.08%, and ~15.22% in the Ctrl, DM, and DM+MRS1754 groups, respectively. Diabetes significantly increased TGF-β1 positivity by ~81% (~1.81-fold) compared with Ctrl animals (*p* = 0.0010). In contrast, A2BAR antagonism significantly reduced TGF-β1 staining by ~44% relative to DM rats (*p* = 0.0011), with no significant difference remaining relative to Ctrl lungs (*p* = 0.9939) (Figure 5G,H). When analyzed by compartment, bronchiolar regions showed values of ~19.76%, ~36.60%, and ~15.98% for Ctrl, DM, and DM+MRS1754, respectively. In this compartment, diabetes significantly increased TGF-β1 positivity by ~85% (~1.85-fold) compared with Ctrl animals (*p* = 0.0003), whereas MRS1754 treatment significantly reduced staining by ~56% relative to DM rats (*p* < 0.0001), effectively normalizing values to the Ctrl group (*p* = 0.4275) (Figure S8). In the vascular compartment, mean percentages were ~14.54%, ~23.60%, and ~16.72% for Ctrl, DM, and DM+MRS1754, respectively. Diabetes significantly increased TGF-β1 positivity by ~62% (~1.62-fold) compared with Ctrl (*p* = 0.0025), while MRS1754 administration significantly reduced positivity by ~29% relative to DM rats (*p* = 0.0160), returning values to a level statistically comparable to Ctrl (*p* = 0.5624) (Figure S8). Finally, in alveolar regions, percentages were ~10.94%, ~22.48%, and ~13.26% for Ctrl, DM, and DM+MRS1754, respectively. In this compartment, diabetes significantly increased TGF-β1 positivity by ~105% (~2.05-fold) compared with Ctrl animals (*p* = 0.0071), whereas MRS1754 treatment significantly decreased staining by ~41% relative to DM rats (*p* = 0.0275), with values once again indistinguishable from Ctrl (*p* = 0.7358) (Figure S8).

Collectively, these findings support an antifibrotic effect of pharmacological A2BAR antagonism with MRS1754 in the lungs of diabetic rats, evidenced primarily by significant DM-induced increases in *Fn1* mRNA, BALF TGF-β1 protein, and compartment-specific TGF-β1 and COL1A1 protein staining, all of which were significantly attenuated by MRS1754 treatment. In contrast, *Acta2* and *Tgfb1* mRNA were paradoxically decreased in DM relative to Ctrl; MRS1754 fully restored *Acta2* mRNA to Ctrl levels but did not affect *Tgfb1* mRNA, a discordance between transcript and protein-level responses that is addressed mechanistically in the Discussion.

### 2.4. Antagonism of A2BAR with MRS1754 Reduces Macrophage Infiltration and Restores M1/M2 Phenotypic Balance in the Lungs of Diabetic Rats

Based on our previous histopathological analysis (Figure 2E), diabetic lungs exhibited abundant mononuclear cell infiltration, morphologically compatible with MΦ. Given that MΦ play central roles in inflammatory responses and fibrotic remodeling [67], we next evaluated whether A2BAR antagonism modulates MΦ accumulation during DLD. To this end, we assessed Cluster of Differentiation 68 (CD68) expression, a well-established pan-MΦ marker [67,68].

**Figure 6:**
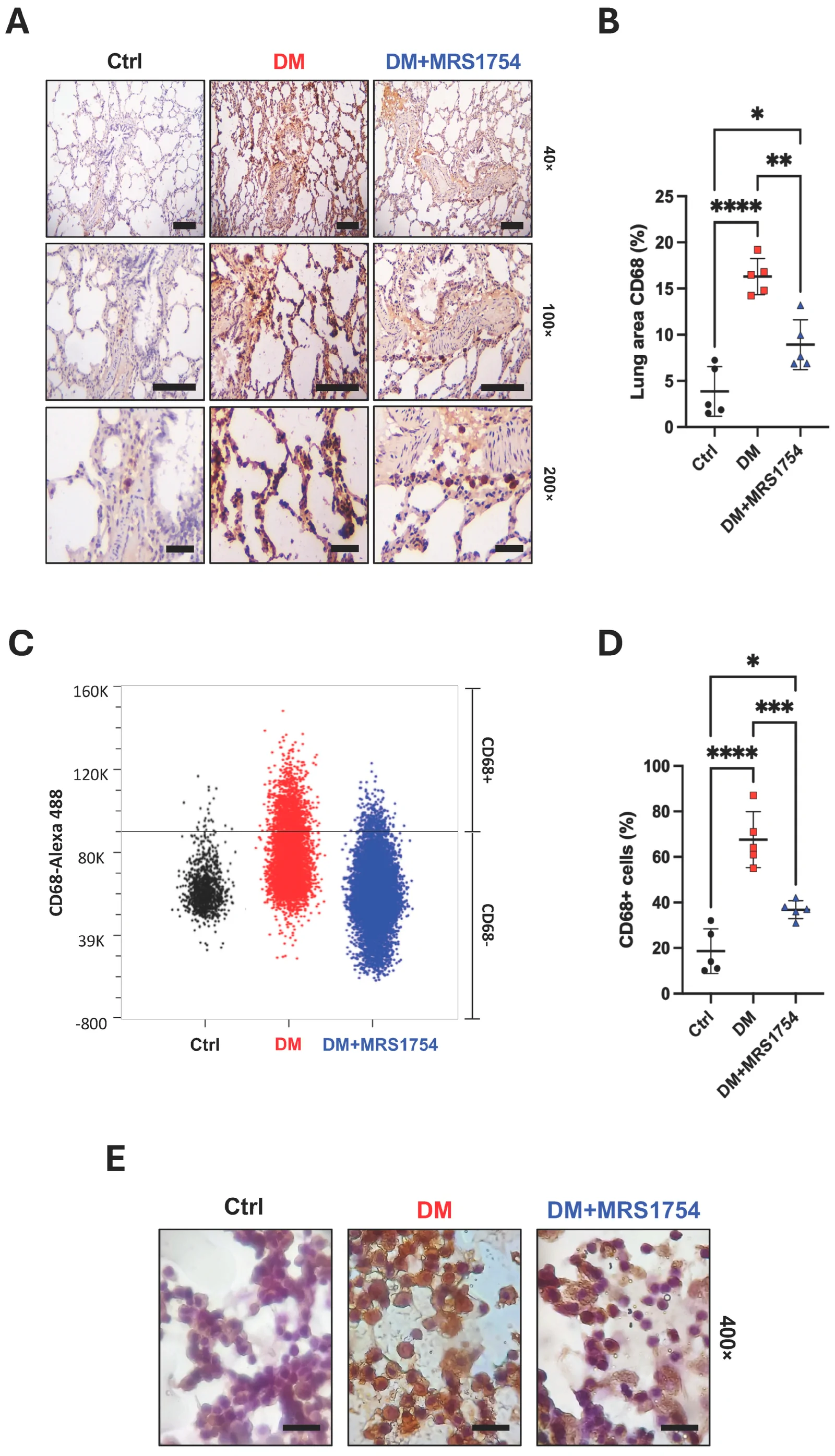
Effect of in vivo MRS1754 treatment on macrophage accumulation in BALF and lung tissue of diabetic rats. (**A**) Representative images of Cluster of Differentiation 68 (CD68) immunohistochemical staining (pan-macrophage marker) in lung sections from Ctrl, DM, and DM+MRS1754 rats. Images show whole lung sections (40× magnification; Scale bar = 100 µm) and higher magnification views at 100× (Scale bar = 100 µm) and 200× (Scale bar = 50 µm), highlighting macrophage (MΦ) infiltration in alveolar septa and peribronchiolar areas. (**B**) Quantitative analysis showing the percentage distribution of CD68^+^ immunoreactivity in whole lung tissue. Seven non-overlapping fields per animal were analyzed and averaged to obtain a single value per animal. Each dot represents one animal (*n* = 5 animals per group). Representative flow cytometry (**C**) scatter plots illustrating CD68-Alexa 488 fluorescence distribution of BALF cells from Ctrl, DM, and DM+MRS1754 rats. (**D**) Flow cytometric quantification of the percentage of CD68^+^ MΦ in BALF. In graph (**D**), each dot represents the mean value obtained per animal (*n* = 5 animals per group). (**E**) Representative images of CD68 immunocytochemistry in BALF from Ctrl, DM, and DM+MRS1754 rats (400× magnification; Scale bar = 20 µm), illustrating MΦ abundance in the alveolar compartment. Graphs are presented as mean ± SD. Statistical analysis was performed using one-way ANOVA followed by Tukey’s multiple comparison test. Statistical significance is indicated as * *p* < 0.05, ** *p* < 0.01, *** *p* < 0.001, and **** *p* < 0.0001; ns indicates no significant difference.

CD68 IHC revealed a significant ~4.21-fold increase in CD68^+^ staining in the lungs of diabetic rats (16.30%) compared with controls (3.868%) (*p* < 0.0001), consistent with enhanced MΦ infiltration (Figure 6A,B). Treatment with MRS1754 (8.920%) significantly reduced the CD68^+^ area by ~45% compared with DM rats (*p* = 0.0013), although CD68^+^ levels in DM+MRS1754 rats remained significantly higher than those in controls (*p* = 0.0183), indicating that in vivo A2BAR antagonism decreased MΦ infiltration in the diabetic lung without fully normalizing it (Figure 6A,B).

In parallel, flow cytometry analysis of BALF cells demonstrated a clear increase in the CD68^+^ population in diabetic rats, evidenced by a rightward shift in CD68-Alexa 488 fluorescence intensity compared with controls (Figure 6C). Quantitative analysis showed that the percentage of CD68^+^ MΦ in BALF was significantly elevated by ~3.63-fold in the DM group (67.60%) compared with Ctrl rats (18.60%) (*p* < 0.0001) (Figure 6D). MRS1754 treatment significantly reduced the percentage of CD68^+^ cells to 36.80%, representing a ~1.84-fold reduction relative to DM rats (*p* = 0.0006), although values in the DM+MRS1754 group remained ~1.98-fold higher than controls (*p* = 0.0244), pointing to a similarly incomplete recovery in the alveolar compartment (Figure 6D).

Consistently, CD68 immunocytochemistry (ICC) of BALF qualitatively confirmed increased MΦ abundance in diabetic rats, which exhibited a foamy/granular cytoplasmic phenotype suggestive of altered MΦ activation and/or intracellular material accumulation. In contrast, both CD68^+^ staining and the foamy/granular cytoplasmic phenotype were reduced in the DM+MRS1754 group (Figure 6E).

Given that MΦ exhibit considerable functional plasticity, polarizing into classically activated M1 (pro-inflammatory/antifibrotic) or alternatively activated M2 (pro-fibrotic/tissue-remodeling) phenotypes depending on the local microenvironment [37,44,45,67,68], we next asked whether the increased CD68^+^ infiltration observed above reflects a specific shift in this phenotypic balance, and whether A2BAR antagonism affects it. To this end, we performed flow cytometry analysis of the M1 (Cluster of Differentiation 86 positive, CD86^+^) and M2 (Cluster of Differentiation 163 positive, CD163^+^) subsets within the CD68^+^ population in BALF.

**Figure 7:**
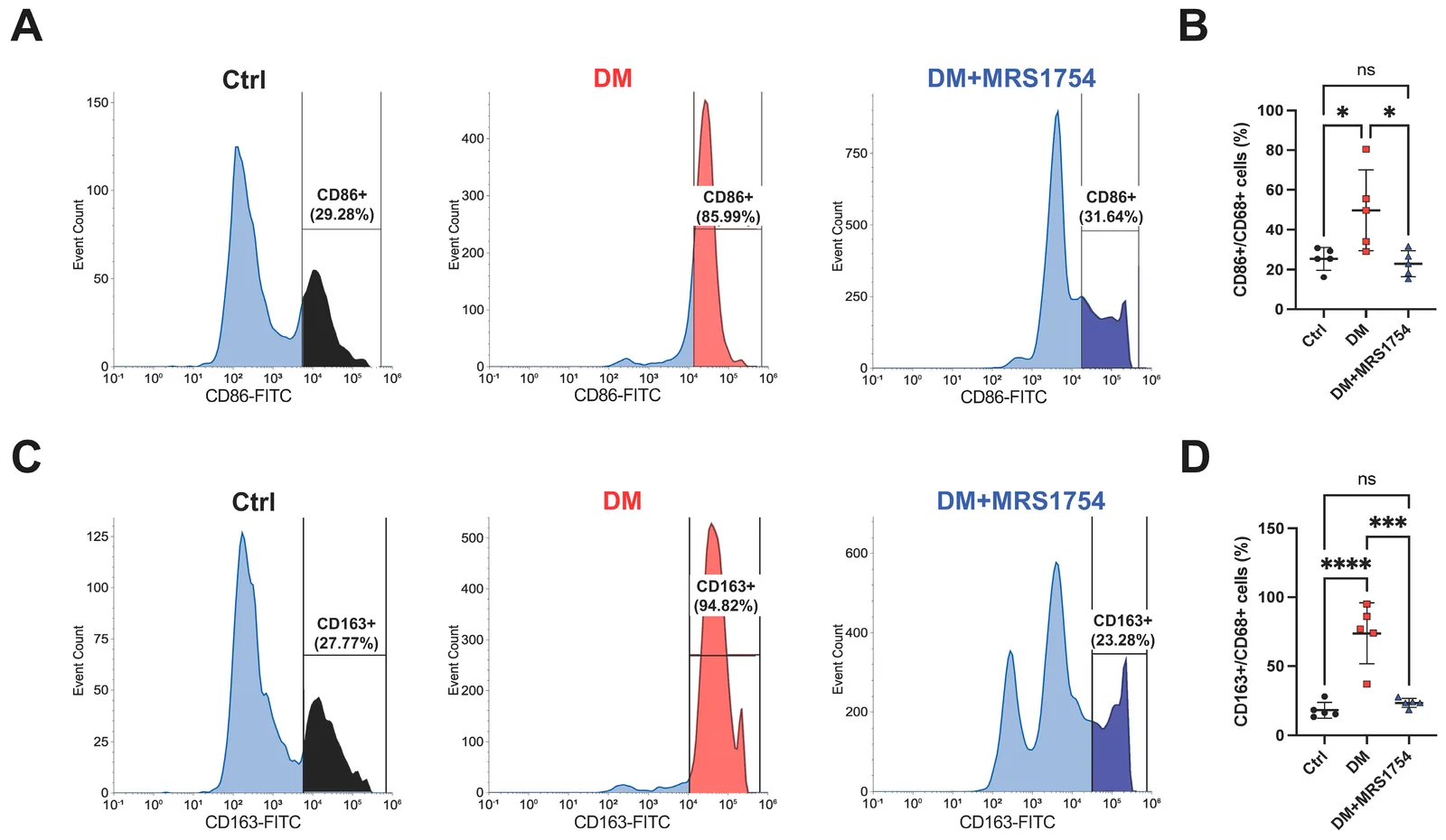
Effect of in vivo MRS1754 treatment on macrophage polarization (M1/M2 balance) in BALF of diabetic rats. Representative flow cytometry histograms illustrating the percentage of (**A**) Cluster of Differentiation 86 positive (CD86^+^; M1, pro-inflammatory) and (**C**) Cluster of Differentiation 163 positive (CD163^+^; M2, pro-fibrotic/tissue-remodeling) cells within the CD68^+^ MΦ population in BALF from control Ctrl, DM, and DM+MRS1754 rats. Gated regions indicate the percentage of positive cells for each representative animal. Quantification of the percentage of (**B**) CD86^+^/CD68^+^ (M1) and (**D**) CD163^+^/CD68^+^ (M2) cells in BALF. Each dot represents the mean value obtained per animal (*n* = 5 animals per group). Graphs are presented as mean ± SD. Statistical analysis was performed using one-way ANOVA followed by Tukey’s multiple comparison test. Statistical significance is indicated as * *p* < 0.05, *** *p* < 0.001, and **** *p* < 0.0001; ns indicates no significant difference.

Both the CD86^+^ (M1) and CD163^+^ (M2) subsets were significantly increased in DM rats compared with controls (CD86^+^: 25.4 ± 5.7% to 49.8 ± 20.3%, a ~1.96-fold increase, *p* = 0.037; CD163^+^: 18.0 ± 5.9% to 73.8 ± 22.1%, a ~4.10-fold increase, *p* = 0.002) (Figure 7). Notably, MRS1754 treatment fully normalized both subsets to levels not significantly different from controls (CD86^+^: 23.0 ± 6.5%, *p* = 0.655 vs. Ctrl; CD163^+^: 23.3 ± 3.4%, *p* = 0.100 vs. Ctrl), representing reductions of ~2.16-fold and ~3.17-fold relative to DM, respectively (both *p* < 0.05 vs. DM) (Figure 7). This indicates that, in contrast to the total CD68^+^ population (Figure 6), the phenotypic balance of infiltrating MΦ is fully restored by A2BAR antagonism, even though overall MΦ numbers remain modestly elevated.

### 2.5. Infiltrating Macrophages Correlate with, and Represent a Major Cellular Source of, the Profibrotic Mediators TGF-β1 and FN1 in Diabetic Lung Disease, Supported by Human Fibrotic Lung Reanalysis

**Figure 8:**
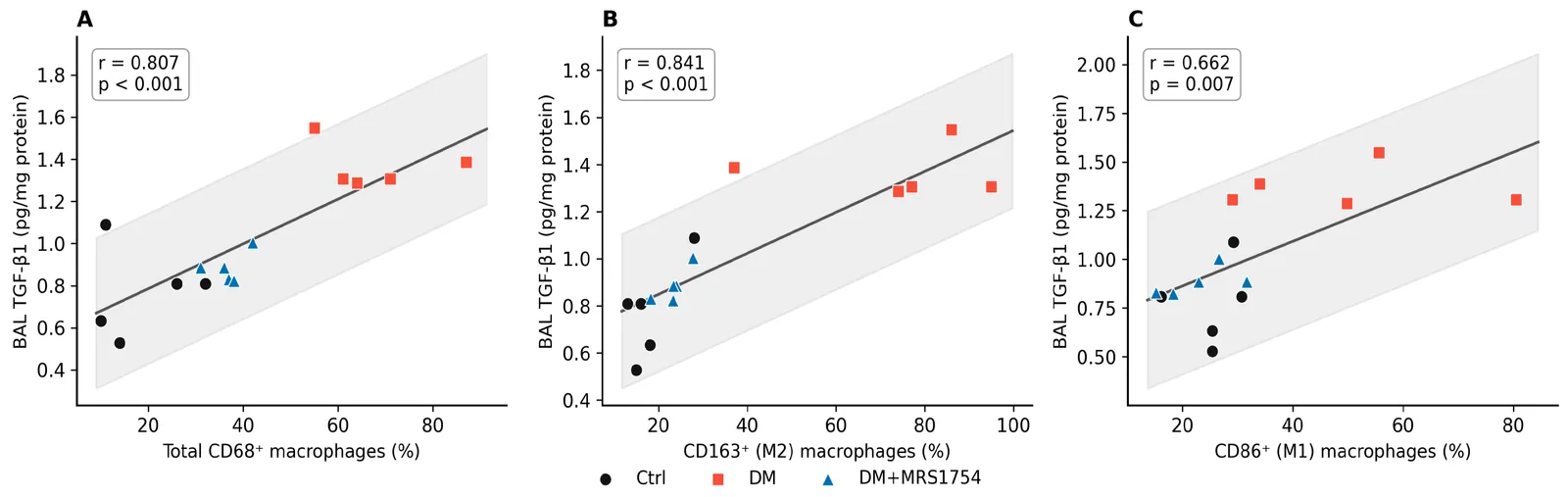
Correlation between bronchoalveolar lavage (BAL) macrophage populations and BAL TGF-β1 levels. (**A**) Total CD68^+^ MΦ (% of total BAL events), (**B**) CD163^+^ (M2, profibrotic) MΦ (% within CD68^+^ population), and (**C**) CD86^+^ (M1) MΦ (% within CD68^+^ population), each plotted against BAL TGF-β1 concentration (pg/mg protein). Data points are pooled from all animals across the three experimental groups (Ctrl, black circles; DM, red squares; DM+MRS1754, blue triangles; *n* = 5/group, *n* = 15 total). Solid lines represent the linear regression fit; shaded bands represent the 95% confidence interval of the fit. Pearson correlation coefficients (r) and *p*-values are shown in each panel.

We then asked whether this MΦ phenotypic shift relates to the BALF TGF-β1 levels reported above (Figure 5D). Both total CD68^+^ (r = 0.807, *p* < 0.001) and, more strongly, CD163^+^ (r = 0.841, *p* < 0.001) percentages positively correlated with BALF TGF-β1, more so than CD86^+^ (r = 0.662, *p* = 0.007) (Figure 8 and Table 2), consistent with a preferential contribution of the M2-like MΦ subset to the profibrotic cytokine milieu, and in line with previous reports identifying CD163^+^/M2-like MΦ as an important source of TGF-β1 and as contributors to FN deposition and ECM remodeling in fibrotic tissues [69].

To extend this observation to human disease, we reanalyzed publicly available single-cell RNA-sequencing data generated from dissociated lung tissue biopsies of transplant donors and lung explants from patients with pulmonary fibrosis of varying etiology (IPF, systemic sclerosis, polymyositis, and hypersensitivity pneumonitis) [49]. In this dataset, MΦ represented the most abundant cell population among all lung-resident cells (40.9% of 76,070 cells; Figure S9A), and *TGFB1*, which encodes TGF-β1, expression was detected in 24.1% of all profiled cells (Figure S9B). Within the MΦ compartment specifically, our reanalysis showed that approximately 43% of MΦ expressed *TGFB1* (Figure S9C); combining both analyses, MΦ accounted for an estimated 69% of all *TGFB1*-expressing cells in this dataset (Figure S9D), providing independent evidence from human fibrotic lung tissue that MΦ are the major cellular source of this profibrotic cytokine. We note that these data derive from whole-lung tissue dissociation rather than BAL, and from a cohort of pulmonary fibrosis of mixed, non-diabetic etiology; as such, they support the general biological plausibility of the MΦ–TGF-β1 axis rather than providing diabetes-specific or BAL-specific validation. Because MΦ identity was defined using two independent clustering approaches across the source analyses, the 69% estimate should be interpreted as approximate rather than as a value directly reported by Reyfman et al. [49].

**Figure 9:**
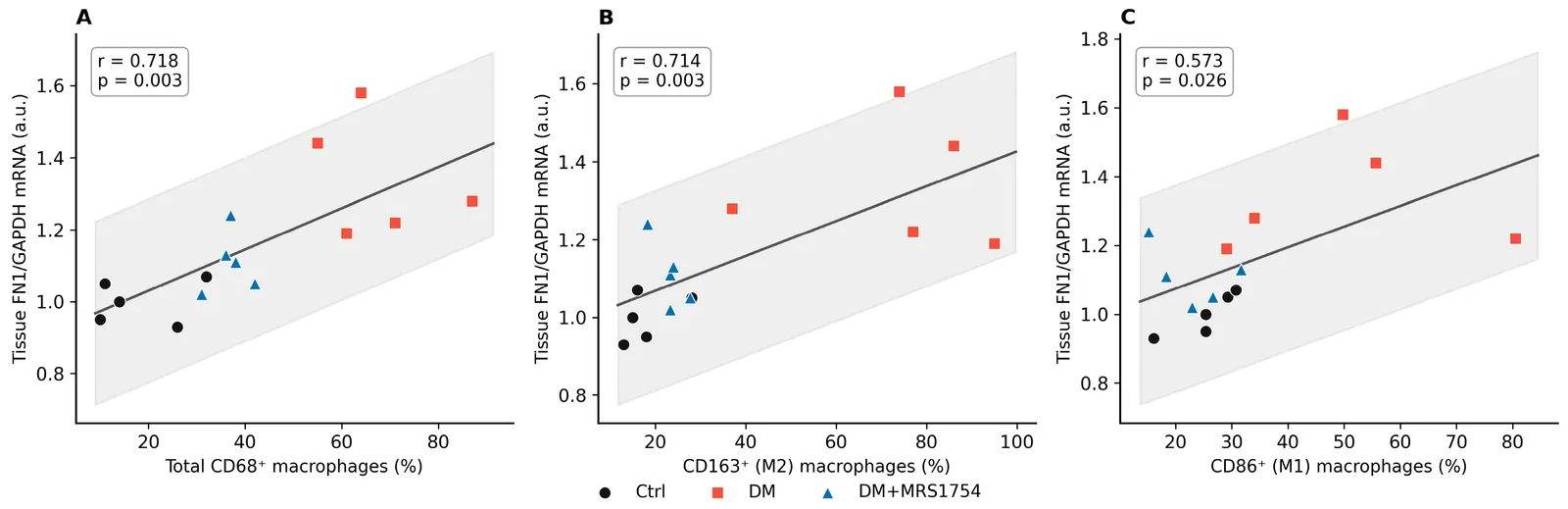
Correlation between BAL MΦ populations and lung tissue *Fn1* mRNA levels. (**A**) Total CD68^+^ MΦ (% of total BAL events), (**B**) CD163^+^ (M2, profibrotic) MΦ (% within CD68^+^ population), and (**C**) CD86^+^ (M1) MΦ (% within CD68^+^ population), each plotted against lung tissue *Fn1*/*Gapdh* mRNA levels (a.u.). Data points are pooled from all animals across the three experimental groups (Ctrl, black circles; DM, red squares; DM+MRS1754, blue triangles; *n* = 5/group, *n* = 15 total). Solid lines represent the linear regression fit; shaded bands represent the 95% confidence interval of the fit. Pearson correlation coefficients (r) and *p*-values are shown in each panel.

Because ECM deposition and fibrosis in this model are also driven by FN, we examined whether *Fn1* levels—previously shown to be increased in the DM group and attenuated by MRS1754 (Figure 5A)—also correlated with MΦ infiltration. *Fn1* mRNA levels correlated positively with BAL CD68^+^, CD163^+^, and CD86^+^ MΦ populations (r = 0.718, *p* = 0.003; r = 0.714, *p* = 0.003; r = 0.573, *p* = 0.026, respectively; Figure 9 and Table 3), paralleling the correlations observed for TGF-β1.

To extend this observation to *FN1* in human disease, we reanalyzed the same dataset [49] for *FN1* expression. *FN1* was detected in 31.9% of all profiled cells (Figure S10A), and within the MΦ compartment specifically, 57.1% of MΦ expressed *FN1* (Figure S10B); combining both analyses, MΦ accounted for an estimated 70% of all *FN1*-expressing cells in this dataset (Figure S10C), providing independent evidence from human fibrotic lung tissue that MΦ are a major cellular source of *FN1* as well as *TGFB1*.

Collectively, these findings indicate that A2BAR antagonism not only attenuates MΦ infiltration but also restores M1/M2 phenotypic balance in the diabetic lung, an effect that correlates with reduced levels of both TGF-β1 and FN1 and is reinforced by independent evidence from human fibrotic lung tissue identifying MΦ as a major cellular source of both mediators. Together, these results support a model in which hyperglycemia/adenosine-driven MΦ recruitment and M1/M2 imbalance sustain local TGF-β1 and FN1 production, which would in turn be expected to drive fibroblast activation and ECM deposition, establishing a self-reinforcing feedback loop between MΦ infiltration and fibrotic remodeling in the diabetic lung; A2BAR antagonism may interrupt this loop by limiting the MΦ-derived profibrotic signal.

## 3. Discussion

This study provides experimental evidence that pharmacological A2BAR antagonism with MRS1754 attenuates key pathological features of DLD in a STZ-induced rat model, including pulmonary inflammation, fibrotic remodeling, and MΦ accumulation. Diabetic lungs displayed pronounced inflammatory infiltration, increased CD68^+^ MΦ burden in lung and BALF, elevated cytokines, enhanced TGF-β1 signaling, and ECM deposition, all mitigated by A2BAR blockade—supporting an association between the adenosine/A2BAR axis and inflammatory/fibrotic responses, although the underlying mechanisms are likely modulatory rather than exclusively causal.

Diabetes is increasingly recognized as a systemic disease with pulmonary involvement, historically overshadowed by renal, retinal, and cardiovascular complications. Diabetes worsens outcomes in COPD patients [70], and diabetic microvascular disease promotes pulmonary inflammatory injury [71] via mechanisms analogous to nephropathy/retinopathy; long-standing type 2 DM patients exhibit a restrictive ventilatory pattern [13] independent of smoking, body mass index (BMI), or glycemic control. Preclinically, STZ-diabetic rats show peribronchial/peribronchiolar immune infiltration quantified with scoring systems analogous to ours [72], and murine type 2 DM models link chronic pulmonary inflammation to alveolar MΦ infiltration [73]. Diabetes can also modulate acute responses: diabetic rats show paradoxically attenuated inflammation after septic injury via impaired NF-κB activation [74], a duality examined below for A2BAR.

Diabetes is associated with a chronic inflammatory lung phenotype with immune infiltration and increased TNF-α, IL-6, and IL-1β in models and patients [10,11,14,15,55], consistent with our findings of marked infiltration, cytokine overexpression, and elevated BALF TNF-α, all significantly attenuated by MRS1754. Extracellular adenosine and A2BAR activation have been linked to amplification of inflammatory mediators and fibrogenesis, including IL-6/TNF-α release, during chronic lung injury [35,36,37,40,41,42,43,46,75,76,77,78,79]. Acute lung injury models, in contrast, report a protective A2BAR role, where blockade can exacerbate inflammation [80,81]. Fibrotic markers (FN, α-SMA, COL1A1, TGF-β1) generally rise during fibrogenesis, yet *Acta2* and *Tgfb1* mRNA were paradoxically decreased in DM rats, while COL1A1 protein showed only a non-significant upward trend—suggesting fibrotic remodeling, though present, may be secondary to inflammation here.

The purinergic adenosine/A2BAR axis is increasingly recognized as a contributor to cardiovascular and metabolic disease, including diabetes [30,31,32,33]. Extracellular ATP released under metabolic/inflammatory stress is rapidly converted to adenosine by CD39/CD73 [34]; the pattern here fits this relationship, as DM rats showed ATP comparable to Ctrl despite markedly elevated adenosine, consistent with enhanced ATP catabolism. Following MRS1754, ATP rose sharply while adenosine returned toward baseline, suggesting A2BAR antagonism may limit adenosine generation, potentially by disrupting an A2BAR-dependent feedforward circuit promoting CD73 expression [82]—a mechanism not yet demonstrated in diabetic lung but plausible given the normalization observed here.

The increase in A2BAR transcript following chronic MRS1754 treatment, without a corresponding further increase in protein (which remained elevated at DM levels), is consistent with a compensatory transcriptional response uncoupled from translation, and/or continued receptor blockade despite unchanged receptor abundance—a pattern previously described for the A2A receptor during prolonged pharmacological antagonism [83]. MRS1754 likely prevents receptor activation rather than reducing abundance, consistent with evidence that A2BAR can be functionally desensitized without changes in mRNA/protein [84]. The two mRNA-protein discordances observed appear mechanistically distinct: in DM, protein rose without a transcript increase, suggesting post-transcriptional regulation; in DM+MRS1754, transcript rose without a further protein increase, potentially reflecting a translational ceiling or a transcription-protein lag. As CD39/CD73 activity, receptor trafficking, and A2BAR turnover were not directly assessed, these mechanisms remain speculative.

Beyond receptor expression, we assessed three canonical A2BAR-associated signaling branches—NF-κB, SMAD3, and Akt1—by Western blot, none showing a consistent or significant pattern across groups, likely reflecting biological variability at this sample size. The NF-κB site assessed, Ser536, is not a straightforward activation marker: high-glucose alone does not increase Ser536 phosphorylation without an additional inflammatory stimulus [85], and this site acts as a negative regulator limiting, rather than amplifying, NF-κB activity [86]—a plausible explanation for the absent pattern. These pathways also do not exhaust the A2BAR signaling repertoire; other nodes or cell type-specific signaling not captured here may participate. We regard the pharmacological loss-of-function evidence—consistent reversal of inflammatory, fibrotic, and macrophage phenotypes by MRS1754—as the primary support for A2BAR engagement, not any single assay.

Diabetic rats displayed a severe histopathological inflammation grade compared with Ctrl, driven by increases across all five criteria: alveolar septal thickness, bronchiolar SM thickness, inflammatory infiltration, vascular congestion, and hyaline membrane formation. MRS1754 did not normalize all features equally: septal thickness, SM thickness, and hyaline membrane formation—a defining feature of diffuse alveolar damage [87]—were restored to Ctrl levels, whereas inflammatory infiltration and vascular congestion, though reduced versus DM, remained elevated. Persistent infiltration may reflect hyperglycemia-driven, A2BAR-independent mechanisms, such as hyaluronan-rich ECM synthesis by airway SM promoting monocyte adhesion [88]. A2BAR antagonism thus differentially modulates distinct components of diabetic lung injury, attenuating but not fully resolving inflammation once established.

Qualitative examination suggested concentric thickening of the pulmonary vessel wall with reduced luminal area in DM lungs, less evident after MRS1754; without morphometry or hemodynamic assessment, no conclusions regarding vascular remodeling or pulmonary hypertension can be drawn. This is noteworthy given that diabetes is associated with more severe pulmonary vascular remodeling in pulmonary arterial hypertension [89], and A2BAR blockade attenuates pulmonary hypertension and vascular remodeling in COPD-associated disease [90]; quantitative measurements are needed to determine whether similar changes occur here.

The inflammatory profile showed a consistent pattern across complementary readouts—*Il6* mRNA, BALF TNF-α protein, and IL-1β/TNF-α protein—with significant DM-induced increases markedly attenuated by MRS1754, generally returning toward Ctrl levels despite differing compartment distributions. This concordance contrasts with the mRNA-protein discordance for TGF-β1, suggesting inflammatory mediators responded more coordinately to A2BAR blockade than the profibrotic response, possibly reflecting the complex multi-level regulation of TGF-β1 signaling [61].

Compartment-specific analysis showed IL-1β and TNF-α did not follow identical spatial patterns. IL-1β showed its largest fold-increase in the bronchiolar compartment of DM rats, where MRS1754 produced the greatest (non-significant) numerical reduction. TNF-α, conversely, was restored to Ctrl levels in bronchiolar and alveolar compartments, with no differences among groups in the vascular compartment—indicating that anti-inflammatory effects of A2BAR antagonism are spatially heterogeneous, depending on cytokine and compartment.

The anti-inflammatory effect of chronic A2BAR antagonism may seem to conflict with in vitro evidence that acute A2BAR activation suppresses MΦ TNF-α production and limits acute injury. This reflects temporal- and disease-stage-dependent A2BAR functions: in the same bleomycin model, A2BAR provides early tissue protection but contributes to persistent inflammatory/fibrotic responses during chronic disease [42], consistent with reviews of context-dependent adenosine receptor signaling [91,92] and a similar duality proposed for idiopathic pulmonary fibrosis [93]. Our 12-week model of sustained hyperglycemia more closely resembles this chronic stage, which may explain why antagonism, rather than activation, was beneficial here.

Beyond inflammation, hyperglycemia activates a profibrotic network in which Advanced glycation end products (AGE)–receptor for advanced glycation end products (RAGE) signaling amplifies reactive oxygen species (ROS) and enhances TGF-β1 signaling [61,62,63,94,95,96,97]; TGF-β1, secreted as a latent complex, requires activation via proteases, integrins, and oxidative stress [62,94,96,97,98,99,100]. Adenosine/A2BAR signaling intersects with this axis, regulating TGF-β1 expression/secretion at the glomerular level in diabetic nephropathy [40,47]—a framework consistent with the signatures observed here.

MTS confirmed increased collagen deposition in diabetic lungs, greatest in the alveolar compartment, followed by peribronchiolar and submucosal regions. MRS1754 significantly attenuated collagen in all three compartments, restoring alveolar/submucosal deposition to Ctrl levels, while peribronchiolar collagen remained slightly (non-significantly) elevated—indicating ECM remodeling responsiveness may differ by compartment.

These findings parallel adenosine deaminase-deficient mice [43,101], where A2BAR antagonism reduced myofibroblast accumulation, α-SMA, collagen, and BALF collagen, with suppressed alveolar MΦ-derived TGF-β1/osteopontin—a plausible explanation for the pronounced alveolar collagen reduction and reduced alveolar CD68^+^ burden observed here. More broadly, adenosine receptor signaling drives fibrosis organ-specifically: A2A predominates in skin, peritoneum, and liver, while A2B predominates in pulmonary fibrosis [102,103], further supporting A2BAR as the relevant target.

Several bronchiolar findings—increased SM thickness, peribronchiolar collagen, and elevated bronchiolar COL1α1/TGF-β1—are conceptually consistent with airway remodeling, established in asthma/COPD [104]. A2BAR is expressed by bronchial smooth muscle, where activation stimulates cytokine release [105], and its inhibition reduces airway remodeling mediators in mice with chronically elevated pulmonary adenosine [43], supporting a role for A2BAR in bronchiolar remodeling here.

Antifibrotic therapies should ideally limit pathological collagen accumulation without compromising ECM’s structural function [106,107,108,109]. Our findings align with interventional studies targeting TGF-β1: baricitinib reduced fibroblast activation via Janus kinase (JAK)/Signal transducer and activator of transcription (STAT) and non-Smad pathways [110], and other TGF-β/Smad-targeting agents diminished collagen [111,112]. ECM homeostasis also depends on the matrix metalloproteinase (MMP)/tissue inhibitor of metalloproteinases (TIMP) system; both diabetic and fibrotic lungs show MMP/TIMP imbalance impairing turnover and crosslinking [113,114,115]—how A2BAR blockade interacts with this system deserves further investigation.

Molecular evaluation revealed a heterogeneous pattern: *Fn1* mRNA was significantly elevated in DM and partially reduced by MRS1754, whereas *Acta2* and *Tgfb1* mRNA were paradoxically decreased, with *Acta2* fully restored by MRS1754; COL1A1 protein showed only a non-significant upward trend. This discordance may reflect genuine biological dissociation, as α-SMA does not consistently correlate with collagen-producing capacity, and myofibroblasts with strong collagen-producing capacity can express relatively low α-SMA in some fibrotic contexts [116], including the lung, where single-cell studies show only a subset of collagen-producing fibroblasts co-express α-SMA [117]. A2BAR stimulation inhibits agonist-induced α-SMA expression via cyclic adenosine monophosphate (cAMP)/exchange protein directly activated by cAMP (Epac)/PI3K/Akt signaling in cardiac fibroblasts [118]; chronically elevated adenosine could tonically suppress α-SMA here, with MRS1754 relieving this suppression—a mechanism not necessarily applicable to TGF-β1, regulated by a broader, partially overlapping network, potentially explaining why its mRNA remained unchanged despite α-SMA normalization.

Notably, whole-lung COL1A1 showed no significant differences, whereas compartment-specific analysis revealed marked alveolar/bronchiolar differences masked by whole-organ averaging, consistent with the methodological advantage of compartment-specific analyses in heterogeneous tissue [119]. Regional differences in fibroblast fibrogenic capacity are similarly documented in human airway disease, where proximal/distal lung fibroblasts differ substantially in collagen synthesis [120], and single-cell studies have identified regional fibroblast subpopulations with distinct TGF-β1-associated SMAD/c-Jun N-terminal kinase (JNK) signaling [121,122]. We hypothesize this heterogeneity may also explain the residual peribronchiolar collagen and absent vascular COL1A1 staining, though not directly tested.

FN exhibited a distinct expression pattern reflecting its cellular source and role in ECM remodeling. Unlike COL1A1 and α-SMA, which primarily reflect mesenchymal activation, FN can be induced by adenosine in lung epithelial cells and fibroblasts via A2BAR-dependent cAMP/cAMP response element-binding protein (CREB) signaling [123]; type II alveolar epithelial cells are the major A2BAR expression site in murine lung, suggesting this population may substantially contribute to the enhanced FN response in diabetic lungs [124]. FN is also highly expressed in injured tissue during repair [123], so its responsiveness to diabetes and MRS1754 may reflect an earlier fibrotic phase than the later collagen-rich matrix represented by COL1A1/α-SMA.

Taken together, these findings do not support a uniform, fully established fibrotic phenotype. Instead, fibrotic remodeling in the diabetic lung appears spatially restricted and heterogeneous at the molecular and histological level, consistent with fibrosis being present but likely secondary to the more consistently observed inflammatory phenotype. As discussed below, this heterogeneous, already-established lesion—rather than an actively progressing one—is the most accurate frame for interpreting the effect of MRS1754.

The treatment window (weeks 4–12 post-STZ; MRS1754 0.5 mg/kg every 48 h, within the effective range reported by Cárdenas et al. (2013) [125]) was deliberately set so treatment began after diabetic lung pathology was already underway, not before its onset (Section 4.1). Our own time-course data show pulmonary collagen deposition already significantly elevated by 1 month post-STZ and unchanged through 3 months, consistent with established pulmonary fibrosis by 6–8 weeks in this model [126] and detectable pulmonary transcriptional changes by 4 weeks post-STZ in diabetic Wistar rats [48]. Because collagen deposition plateaus rather than progresses in this window untreated, the reduction with MRS1754 cannot represent prevention of an ongoing process; it more accurately reflects attenuation of an already-established, stable lesion. Although the 1- to 3-month difference was not significant, a modest upward trend was observed; we cannot exclude slower progression beyond this window, and the natural history of diabetic pulmonary fibrosis beyond the studied period remains uncharacterized.

MΦ accumulation represents a critical component of the DLD phenotype. CD68^+^ infiltration in lung and BALF was significantly reduced by MRS1754, though not fully normalized. MΦ are key orchestrators of chronic inflammation and fibrotic remodeling, contributing to cytokine production, TGF-β1 activation, and ECM turnover [37,67]; in fibrotic lung diseases such as IPF, MΦ accumulation drives TGF-β-dependent profibrotic responses and disease progression [43,127].

Extracellular adenosine regulates MΦ recruitment/activation via A2BAR-dependent mechanisms promoting pro-inflammatory/profibrotic signaling. MRS1754 similarly reduced MΦ infiltration and attenuated renal fibrosis in experimental diabetic nephropathy [36,37], suggesting A2BAR-mediated MΦ accumulation may be a shared pathogenic mechanism across diabetic organs, though causality cannot be established here.

MΦ polarization was directly assessed via flow cytometric CD86^+^ (M1) and CD163^+^ (M2) quantification in BALF. Both subsets increased significantly in DM, with CD163^+^ (M2, profibrotic) expanding more (~4.1-fold) than CD86^+^ (M1, ~2.0-fold), indicating a shift toward M2 predominance; MRS1754 fully normalized both subsets. This is consistent with reports that A2A/A2B upregulation promotes M2-like activation [45,128] and complements our previous work showing A2BAR antagonism decreases pro-fibrotic M2 polarization in diabetic nephropathy [37], suggesting a shared mechanism across diabetic organs.

**Figure 10:**
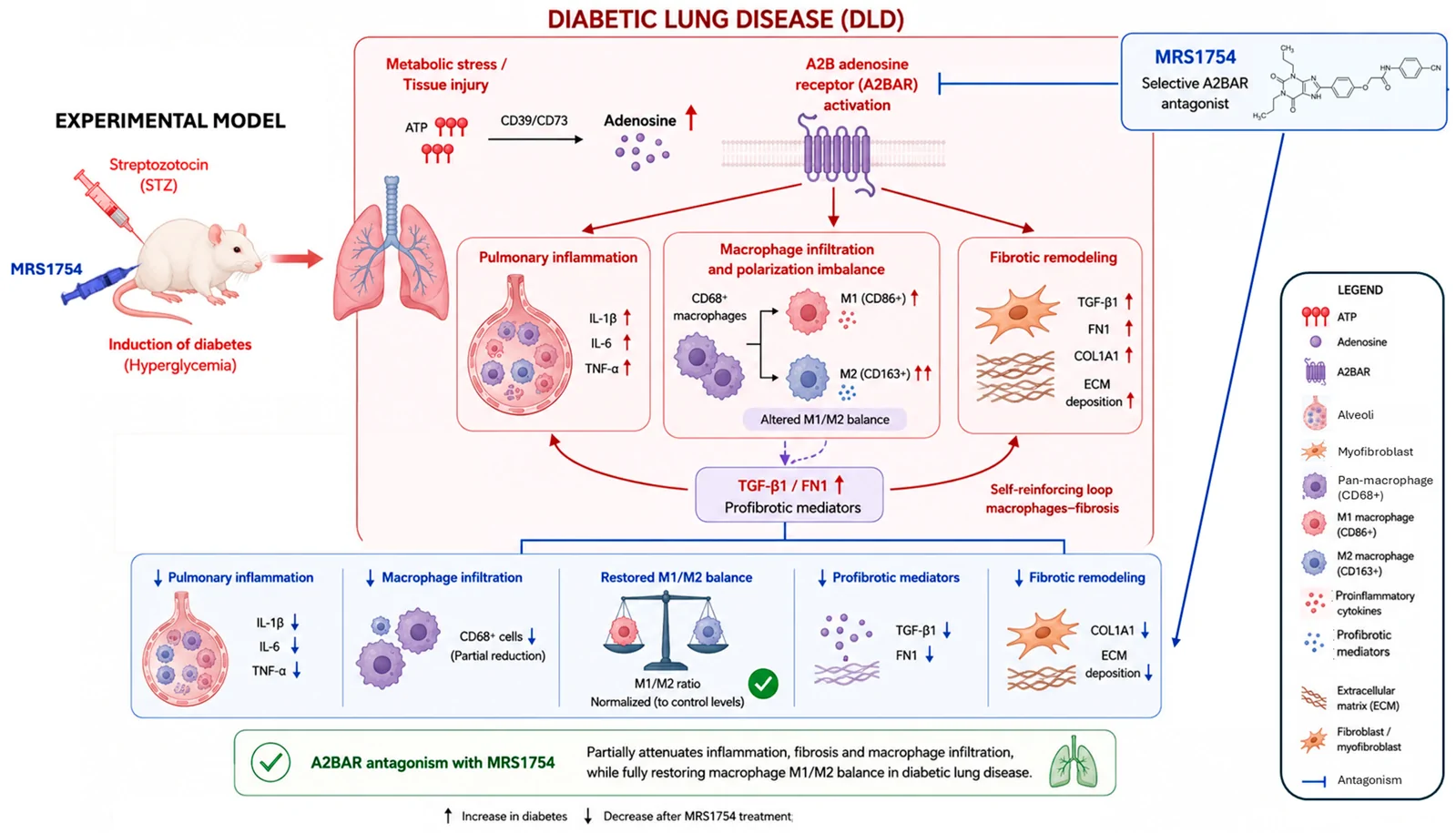
Proposed model of diabetes-associated pulmonary alterations mediated by the extracellular adenosine axis, and their modulation by A2BAR antagonism with MRS1754. STZ-induced diabetic rats were treated with the selective A2BAR antagonist MRS1754. Diabetes-associated metabolic stress and tissue injury increase extracellular ATP, which is hydrolyzed to adenosine via the ectonucleotidases CD39/CD73, driving A2BAR activation in the lung. A2BAR engagement promotes three convergent pathological processes: (i) pulmonary inflammation, with increased IL-1β, IL-6, and TNF-α; (ii) MΦ infiltration and a polarization imbalance within the CD68^+^ population, with disproportionate expansion of the M2 (CD163^+^) relative to the M1 (CD86^+^) subset; and (iii) fibrotic remodeling, with increased TGF-β1, FN1, COL1A1, and ECM deposition. Profibrotic mediators released by infiltrating MΦ (TGF-β1, FN1) further reinforce both the inflammatory and fibrotic compartments, establishing a self-reinforcing MΦ–fibrosis loop. Pharmacological blockade of A2BAR with MRS1754 partially attenuates pulmonary inflammation, MΦ infiltration, and fibrotic remodeling, while fully restoring the M1/M2 MΦ balance to levels not significantly different from non-diabetic controls. Arrows within the DLD panel (↑) indicate diabetes-induced increases; arrows within the MRS1754 treatment panel (↓) indicate reductions following treatment, relative to untreated diabetic animals.

Because the M2-like subset expanded most prominently and correlated strongly with BALF TGF-β1 and lung *Fn1* mRNA, we propose MΦ—particularly M2-polarized MΦ—as a central cellular link between diabetes-driven inflammation and fibrotic remodeling. Hyperglycemia/adenosine-driven MΦ recruitment and M1/M2 imbalance would sustain local TGF-β1/FN1 production, activating resident fibroblasts and driving ECM deposition in a self-reinforcing MΦ-fibrosis loop (Figure 10); A2BAR antagonism may interrupt this loop. This is supported by our human single-cell reanalysis, where MΦ were the most abundant lung-resident population and major source of *TGFB1*- and *FN1*-expressing cells, together with compartment-specific IHC patterns consistent with contributions from bronchiolar SM/epithelium and alveolar MΦ/type II epithelial cells, both A2BAR expression sites [43,105,124]—informative regarding which cell populations are engaged, though not causality.

Establishing cell-autonomous causality would require direct genetic or pharmacological manipulation of A2BAR in specific cell types—alveolar epithelial cells, resident fibroblasts, or bone marrow-derived MΦ—via cell type-specific deletion or agonist/antagonist rescue in primary cells or organoids, none performed here. Branching human iPSC-derived lung organoids, in parallel development by our group, offer a feasible future platform for this.

An additional methodological consideration concerns marker selection: CD68/ED1 is the validated pan- MΦ marker for rat lung [67,68] and was used here. Although CD68 has been reported to also recognize activated fibroblasts in fibrotic mouse tissue, F4/80 more specifically distinguished MΦ there but is not validated in rat [129]; thus, while most CD68^+^ cells likely represent MΦ, a small proportion of fibrotic-region signal may originate from activated fibroblasts.

A2BAR is a pharmacologically tractable target with prior clinical experience: CVT-6883 completed Phase I evaluation with efficacy in preclinical models of pulmonary inflammation and airway hyperresponsiveness [43,130,131], and PBF-1129 more recently completed a Phase I trial in non-small-cell lung cancer with a favorable safety profile and relevant receptor inhibition [132]. Although development has shifted toward oncology, these studies support the feasibility of A2BAR antagonism in this previously unexplored indication.

Limitations include the STZ-induced type 1 diabetes model, small sample size, absence of pulmonary functional assessment, and pharmacological inhibition, which cannot exclude off-target effects. A formal dose–response analysis was not performed in the lung; the selected dose was supported by our previous studies in this model and other diabetic organs, but the optimal pulmonary dose cannot be definitively established here. Inflammatory cytokines were assessed using distinct, complementary methods by cytokine/compartment (*Il6* by qPCR; TNF-α by BALF ELISA and IHC; IL-1β by IHC only), reflecting biological rationale and the constraint of non-overlapping BALF/biopsy subsets; this limits cross-marker comparison, and complementary mRNA/protein data were not generated for each marker. Hydroxyproline quantification—the gold-standard biochemical measure of pulmonary collagen—was also not performed, as no additional tissue was reserved for this assay beyond that used for histology (FFPE) and Western blot; we consider it a priority for future work.

Several proposed mechanisms were not directly investigated, including CD39/CD73 activity and cAMP/Epac/PI3K/Akt-mediated regulation of α-SMA. Pulmonary vascular remodeling was assessed only qualitatively, FN only at the whole-lung mRNA level, and CD68 labeling of activated fibroblasts was not formally excluded. Cell-autonomous causality for A2BAR effects in specific pulmonary cell types was also not established, as discussed above.

Future studies should determine whether these findings extend to type 2 DM models and establish functional relevance via respiratory mechanics and quantitative vascular morphometry/pulmonary arterial pressure assessment. Mechanistically, future work should extend the cytokine panel to chemokines, IL-10, and NF-κB activation status; incorporate spatial/additional-marker M1/M2 characterization; exclude potential CD68 fibroblast labeling; directly quantify CD39/CD73 activity; evaluate Smad2/3 and JAK/STAT signaling; and pursue cell type-specific causal approaches alongside longitudinal ECM turnover and compartment-specific analyses to define regional fibrotic heterogeneity.

## 4. Materials and Methods

### 4.1. Animals and Sample Biopsies

Male Sprague-Dawley rats aged 6 weeks and weighing 200–250 g were divided into three groups: (1) non-diabetic control (*n* = 10), (2) diabetic (*n* = 10), and (3) diabetic treated with MRS1754 (*n* = 10). Rats were housed in acrylic cages (40 × 20 × 20 cm; length × width × height) with paper pellet bedding, provided with chow and water ad libitum, and maintained under a 12 h light/12 h dark cycle in a temperature-controlled and ventilated animal facility (18–22 °C). Environmental enrichment was provided using acrylic tubes and nesting materials. Animal welfare was monitored throughout the experiment, and animals showing signs of severe distress were humanely euthanized in accordance with the AVMA Guidelines for the Euthanasia of Animals (2020) [133].

To induce DM, rats received a single intravenous injection of STZ at a dose of 65 mg/kg (#1621, Tocris Bioscience, Bristol, UK) [35,36,37,40]. Rats from the Ctrl were injected with an equivalent volume of STZ vehicle (citrate buffer pH 4.5). One week after injection, all animals were weighed, blood glucose levels were measured, and DM was confirmed by observing glycemia levels between 250–400 mg/dL along with proteinuria and glucosuria (Table S2) [35,36,37,40]. Four weeks after STZ injection, a subset of DM rats was treated with intraperitoneal injections of MRS1754 (DM+MRS1754 group; 0.5 mg/kg every 48 h; #2752, Tocris Bioscience, Bristol, UK) [35,36,37,40] for a period of eight weeks. Control DM rats received an equivalent volume of the MRS1754 vehicle (1× phosphate-buffered saline, 1× PBS; #BM-1340, Winkler, Santiago, Chile). At week twelve post-STZ injection, all rats were euthanized by overdose of isoflurane (#10019036060; Baxter, Deerfield, IL, USA) [133,134,135] and weight, glycemia, proteinuria and glucosuria were measured (Table S2).

The MRS1754 dose (0.5 mg/kg) and 48 h administration interval were selected based on our previous studies in the same STZ-induced diabetic rat model [35,36,37,40], in which this regimen consistently produced significant A2BAR-dependent effects, and were kept unchanged here to preserve comparability across target organs. This dose lies within the effective range reported by Cárdenas et al. (2013), who found no significant difference in efficacy between 0.2 and 1.0 mg/kg MRS1754 in a model of diabetic glomerulonephritis [125]. The onset (4 weeks) and duration (8 weeks; 12-week/3-month total study) of treatment were determined based on the established timeline of diabetic organ damage in our model, in which renal damage is evident by 1 month and clearly manifests by 3 months post-STZ. To confirm that pulmonary damage follows a comparable timeline, we performed a time-course analysis of pulmonary collagen deposition (Masson trichrome stain area) in non-diabetic controls and at 1, 2, and 3 months post-STZ injection; collagen deposition was already significantly elevated by 1 month post-STZ and did not increase further through 3 months (Figure S11), consistent with independent reports showing that pulmonary fibrosis in STZ-induced diabetic rats is established by 6–8 weeks post-induction [126]. Treatment was therefore initiated at 4 weeks—once diabetes and early pulmonary fibrotic remodeling were already established—and continued to the 12-week endpoint, at which pulmonary damage is stably manifest.

Half of the animals in each group underwent BAL, and the remaining animals were allocated to lung tissue biopsy; BAL and lung biopsy are mutually exclusive terminal procedures, as BAL disrupts pulmonary architecture and cellular composition and precludes subsequent histological and immunohistochemical analysis of the same lung. In addition, BAL fluid was used for multiple downstream analyses (quantification of ATP, adenosine, and cytokines by ELISA, flow cytometry analysis, and ICC), which required an adequate, undiluted sample volume and cell yield per animal; combining BAL collection with biopsy procedures in the same animals would have compromised both tissue quality for histology and BAL volume/quality for these assays (Figure 1A). Lung tissue samples were consistently collected from the left lobe and the right cranial lobe in all animals across all experimental groups, in accordance with standard rodent lung sampling guidelines [136]. Then, lung tissues were fixed in ice-cold 4% paraformaldehyde (PFA; #28906, Thermo Fisher Scientific, Waltham, MA, USA), paraffin-embedded, and sectioned for subsequent histological and immunohistochemical analyses, including hematoxylin and eosin staining (H&E), MTS, IHC, and RT-qPCR (Figure 1A).

All procedures were carried out in accordance with the National Institutes of Health Guide for the Care and Use of Laboratory Animals, 8th edition (2011) (http://grants.nih.gov/grants/olaw/guide-for-the-care-and-use-of-laboratory-animals.pdf (accessed on 26 August 2026)) and approved by the Institutional Committee on the Use of Live Animals in Research at the Universidad Austral de Chile (approval number 309/2018) and at the Universidad de Talca (approval number N°2023-03-A-E2). All possible measures were taken to minimize animal suffering and to reduce the number of animals used.

### 4.2. Bronchoalveolar Lavage (BAL)

The BAL was performed following the protocol described by Van Hoecke and collaborators with slight modifications [137]. Once euthanasia has been performed by overdose of isoflurane, the animal was positioned on a glass tray. With the help of a 23 G needle, the trachea was pierced and then a 22 G cannula was inserted through the perforation, up to 0.5 cm into the trachea. Later, 3 mL of EDTA saline buffer (0.9% NaCl, 100 µM EDTA; #501745, Winkler, Santiago, Chile) supplemented with a protease inhibitor cocktail (#S8830, Sigma-Aldrich, Darmstadt, Germany) was injected and slowly collected by gently massaging the chest. This procedure was repeated twice, collecting the liquid in a 15 mL conical tube, which was centrifuged at 400× *g* for 7 min at 4 °C. The supernatant was recovered and stored at −80 °C, while the pellet was resuspended in 0.2 mL of erythrocyte lysis buffer (#00-4333-57, Thermo Fisher Scientific, Waltham, MA, USA) for 2 min at room temperature and then centrifuged for 7 min at 400× *g*. The collected supernatant or BALF was used to quantify ATP, adenosine, TNF-α, and TGF-β1.

### 4.3. Adenosine Triphosphate (ATP) Measurement in BALF

ATP levels in BALF were quantified using a luminescence-based ATP Detection Assay Kit (#700410, Cayman Chemical, Ann Arbor, MI, USA) following the manufacturer’s instructions and as previously described by Chen et al. [138], with minor modifications. Briefly, BALF samples were centrifuged at 10,000× *g* for 5 min at 4 °C to remove cellular debris, and supernatants were kept on ice until analysis. Samples were diluted in 1× ATP Detection Sample Buffer when necessary to ensure that luminescence values fell within the linear range of the standard curve. The reaction mixture was freshly prepared by combining 1× ATP Detection Assay Buffer with D-luciferin and luciferase immediately before use. For each well of a white 96-well plate, 100 µL of reaction mixture and 10 µL of sample or ATP standard were added (final volume: 110 µL). After incubation for 15–20 min at room temperature protected from light, luminescence was measured using a Synergy™ H1 Hybrid Multi-Mode Microplate Reader (BioTek Instruments, Winooski, VT, USA). ATP concentrations were calculated from a standard curve generated by serial dilution of the provided ATP standard and then were normalized by protein content of BALF. Each sample was measured in duplicate.

### 4.4. Adenosine Measurement in BALF

Adenosine levels in BALF were quantified by high-performance liquid chromatography (HPLC) with fluorescent detection following 2-chloroacetaldehyde derivatization, as previously described [139], with minor modifications. Briefly, BALF samples were centrifuged at 10,000× *g* for 5 min at 4 °C to remove cellular debris. An aliquot of 200 µL of the supernatant was mixed with 100 µL of citrate buffer (pH 4.0) and subjected to derivatization with 2-chloroacetaldehyde (#35264, Sigma-Aldrich, Darmstadt, Germany) to generate the fluorescent 1,N6-etheno-adenosine derivative. Samples were incubated at 60 °C for 45 min and subsequently cooled on ice prior to analysis. Adenosine was separated using a Chromolith^®^ Performance RP-18e column (#1520010001, Merck, Darmstadt, Germany) and detected by fluorescence (excitation 260–280 nm; emission 410–420 nm). Quantification was performed using external adenosine standards processed under identical derivatization conditions. Adenosine concentrations were normalized to protein content in BALF samples. Each sample was measured in duplicate.

### 4.5. SDS-PAGE and Western Blot

To evaluate A2BAR downstream signaling, 60 μg of protein extracted from lung tissue was separated by 10% SDS-PAGE and transferred onto 0.45 μm PVDF membranes (#10600023, Amersham, Buckinghamshire, UK). Membranes were blocked using a solution of 1× TBS (#BM-1950, Winkler, Santiago, Chile), 0.1% Tween-20 (#BM-2031, Winkler, Santiago, Chile), and 3% bovine serum albumin (BSA; #BM-0150, Winkler, Santiago, Chile), and then incubated with primary antibodies specific for phospho-Ser423/425-SMAD3, total SMAD3, phospho-Ser536-NF-κB p65, total NF-κB p65, phospho-Ser473-Akt1, total Akt1, and β-actin (ACTB) (Table S3). Membranes were first probed for each phospho-specific antibody, stripped using a mild stripping buffer, and reprobed for the corresponding total protein on the same membrane to allow direct calculation of phospho/total ratios. Detection was performed with horseradish peroxidase (HRP)-conjugated secondary antibodies and visualized using SuperSignal™ West Pico Plus Chemiluminescent Substrate (#34577, Thermo Fisher Scientific, Waltham, MA, USA). Images were captured using the Syngene G:Box system (Synoptics Ltd., Cambridge, UK) and analyzed by densitometry with ImageJ software, version 1.54g (National Institutes of Health, Bethesda, MD, USA). Phosphorylated protein levels were normalized to their respective total protein, and total protein levels were normalized to ACTB. Finally, the data are normalized to 1 based on the average of the control group.

### 4.6. ELISA in BALF

The protein levels of TNF-α and TGF-β1 in BALF were measured by Rat TNF-α (ELK1396; ELK Biotechnology, Wuhan, China) and TGF-β1 (ELK2311; ELK Biotechnology) ELISA Kits according to the manufacturer’s instructions. Briefly, samples and cytokine standards were added to the wells and incubated at 37 °C for 80 min. Then the wells were washed and incubated with the biotin-conjugated antibody solution at 37 °C for 50 min. Next, the wells were washed and incubated with the Streptavidin-HRP solution at 37 °C for 50 min. Afterward, the wells were washed and incubated with the 3,3′,5,5′-tetramethylbenzidine (TMB) Substrate Solution at 37 °C for 20 min in the dark. Finally, the stop solution was added, and the absorbance was determined at 450 nm by a microplate reader (AMR-100T; Hangzhou Allsheng Instruments, Hangzhou, China). Results were plotted against the linear portion of the standard curve and then normalized by protein content of BALF. Each sample was measured in duplicate.

### 4.7. Flow Cytometry

Cells obtained from the BAL were evaluated by flow cytometry (BD FACS Melody™ cell sorter, BD Biosciences, San Jose, CA, USA). Briefly, 1.0 × 10^6^ cells were fixed with 3.7% PFA for 15 min at room temperature and permeabilized with cold methanol for 45 min at 4 °C. Then, nonspecific binding of the primary antibody was blocked for 45 min with 1× PBS-BSA 0.5% at room temperature [140]. Finally, cells were marked with the primary antibodies anti-CD68 (ab125212, Abcam, Cambridge, UK), anti-CD86/B7-2 (SC-28347, Santa Cruz Biotechnology, Santa Cruz, CA, USA), and anti-CD163 (SC-58965, Santa Cruz Biotechnology, Santa Cruz, CA, USA) at 4 °C overnight and then detected by flow cytometry using their respective secondary antibody Alexa Fluor 488 and Alexa Fluor 568 (Table S3).

### 4.8. Immunocytochemistry (ICC)

BALF cell pellets were adhered to coverslips using a Poly-L-Lysine Cell Attachment Protocol [139], fixed with 3.7% PFA for 10 min, and permeabilized with 1× PBS containing 0.1% Triton^®^ X-100 (#112298; Merck/Supelco, Darmstadt, Germany) for 10 min. Cells were blocked with 2.5% normal horse serum (S-2012-50, Vector Laboratories, Newark, CA, USA) and 1% BSA (blocking solution) in 1× PBS (blocking solution), followed by overnight incubation at 4 °C with a primary anti-CD68 antibody (ab125212, Abcam, Cambridge, UK) diluted in blocking solution. After washing, cells were incubated for 45 min at room temperature with the ImmPRESS^®^ HRP Horse Anti-Rabbit IgG Polymer Detection Kit, Peroxidase (MP-7401-15, Vector Laboratories, Newark, CA, USA). Immunoreactivity was visualized using the ImmPACT^®^ DAB Substrate, Peroxidase (SK-4105, Vector Laboratories, Newark, CA, USA). Nuclei were counterstained with hematoxylin, and slides were mounted using Histomount™ mounting medium (HS-103, National Diagnostics, Atlanta, GA, USA). Images were acquired at 4×, 10×, and 40× magnification using a bright-field microscope (Olympus^®^, model CX23, Tokyo, Japan).

### 4.9. Hematoxylin and Eosin

After mounting on silanized slides, the 5 μm thick sections were deparaffinized with xylene and rehydrated in a series of solutions of decreasing ethanol concentration (100%, 95%, 70%, and 50% ethanol). The samples were then stained with H&E to later be dehydrated in a series of solutions of ascending ethanol concentration (50%, 70%, 95% and 100% ethanol), ending with xylene, and the slides were then mounted using Histomount^TM^ mounting medium (HS-103, National Diagnostics, Atlanta, GA, USA).

Images were captured using a bright-field microscope (Olympus^®^, model CX23) at 10× and 40× objectives, sampling the left lung lobe following a systematic random pattern across all available quadrants and yielding between 15 and 20 images per slide that met pre-defined quality criteria (adequate staining contrast, absence of tissue folding/tearing, and no field dominated by large airways or vessels). A lower number of qualifying fields was more frequently obtained in DM animals due to greater tissue fragility and more advanced fibrotic remodeling in this group. Lung tissue architecture was evaluated using a histopathological damage score based on the following criteria: (1) thickness of alveolar septa; (2) bronchiolar smooth muscle thickness; (3) infiltration of inflammatory cells; (4) congestion in blood vessels; and (5) hyaline membrane formation [141,142].

Each criterion was classified on a scale of 0 to 3, where: 0 meant without change; 1 meant a mild change; 2 meant a moderate change; 3 meant that severe changes were found. The addition of the scores allowed the classification of the degree of lung inflammation as follows: 0–3: no inflammation; 4–7: mild inflammation; 8–11: moderate inflammation; 12–15: severe inflammation.

### 4.10. Masson Trichrome Staining

Lung biopsies were processed at the pathology laboratory of the University of Talca, Chile, following the instructions of a commercial MTS kit (#100485, Sigma-Aldrich, Darmstadt, Germany).

Once the samples were stained, the distribution of the staining was visualized with the 10× and 40× objectives of a brightfield microscope (Olympus^®^, model CX23), capturing, in a systematic random manner across all available quadrants of the left lung lobe, between 15 and 20 images per slide that met pre-defined quality criteria (adequate staining contrast, absence of tissue folding/tearing, and no field dominated by large airways or vessels); a lower number of qualifying fields was more frequently obtained in DM animals due to greater tissue fragility and more advanced fibrotic remodeling in this group. Finally, the images were processed and analyzed with ImageJ software (National Institutes of Health, Bethesda, MD, USA) using the deconvolution property.

### 4.11. Modified Ashcroft Scoring

Pulmonary fibrosis severity was additionally assessed using the modified Ashcroft scale (0–8) [143,144], applied to both H&E and Masson’s trichrome-stained lung sections described in Section 4.9 and Section 4.10, respectively. For each slide, 25 non-overlapping fields were captured in a systematic random manner across all available quadrants of the left lung lobe, using the 4×, 10×, and 40× objectives of a brightfield microscope (Olympus^®^, model CX23).

Each field was scored by a single observer (Á.T.-A.), blinded to experimental group assignment, based on septal thickening, cellular infiltration, and architectural distortion, following the criteria described by Hübner et al. [144]. For each animal, a single mean Ashcroft score was calculated by averaging scores across all 25 evaluated fields.

### 4.12. Immunohistochemistry (IHC)

The histopathological lung samples were processed at the pathology laboratory of the University of Talca, where they were paraffin-embedded and mounted on silanized slides. Afterward, the 5 μm thick sections were deparaffinized with xylene and rehydrated in a series of solutions of decreasing ethanol concentration (100%, 95%, 70%, and 50% ethanol). Heat-mediated antigen retrieval was performed in citrate buffer (10 mM sodium citrate, 0.05% Tween 20, pH 6.0) by heating it in the microwave every 5 min for half an hour and subsequently, samples were cooled at room temperature for 30 min. For IHC, samples were incubated with BLOXALL^®^ Blocking solution (SP-6000; Vector Laboratories, Newark, CA, USA) for 15 min. For blocking, 2.5% normal horse serum (S-2012-50; Vector Laboratories, Newark, CA, USA) and 1% BSA were used for 30 min each. Immunodetections were performed using primary antibodies: anti-A2BAR, anti-IL-1ꞵ, anti-TNF-α, anti-COL1A1, anti-TGF-ꞵ1, and anti-CD68 (Table S3) in blocking solution overnight at 4 °C [140]. Then samples were incubated with ImmPRESS HRP Horse Anti-Rabbit IgG Polymer Detection Kit, Peroxidase (MP-7401-15, Vector Laboratories, Newark, CA, USA) for 45 min at room temperature and immunosignals were visualized with the use of the ImmPACT^®^ DAB Substrate, Peroxidase (SK-4105; Vector Laboratories, Newark, CA, USA). Counterstaining of nuclei was performed by applying hematoxylin classic stain, followed by mounting the slides using Histomount^TM^ mounting medium (HS-103, National Diagnostics, Atlanta, GA, USA).

Negative controls, performed by omission of the primary antibody (secondary antibody only), are shown in Figure S12.

Images were captured from the left lung lobe following a systematic random pattern across all available quadrants using a bright-field microscope (Olympus^®^, model CX23) with 4×, 10×, 20× and 40× objectives, yielding between 15 and 20 images per slide that met pre-defined quality criteria (adequate staining contrast, absence of tissue folding/tearing, and no field dominated by large airways or vessels). A lower number of qualifying fields was more frequently obtained in DM animals due to greater tissue fragility and more advanced fibrotic remodeling in this group. Images were analyzed using color deconvolution with ImageJ software (National Institutes of Health, Bethesda, MD, USA).

### 4.13. RNA Isolation and RT-qPCR

Total RNA was isolated from formalin-fixed paraffin-embedded (FFPE) rat lung extracts using a Presto^TM^ FFPE RNA Mini Kit according to the manufacturer’s instructions (RF050, Geneaid Biotech, New Taipei City, Taiwan). An additional homogenization step was included before crosslinking reversal utilizing QiaShredder columns (#79656, QIAGEN^®^, Germantown, MD, USA). Subsequently, isolated total RNA (1 µg) was used to synthesize cDNA using the High-Capacity cDNA Reverse Transcription Kit with the MultiScribe Reverse Transcriptase (#4368814, Applied Biosystems, Foster City, CA, USA). qPCRs reactions were performed in an Mx-3000P real-time PCR thermal cycler Stratagene^®^ (Agilent Technologies, Santa Clara, CA, USA) using SYBR green Brilliant Blue III reagent (#600882, Agilent Technologies, Santa Clara, CA, USA) and gene-specific primers targeting rat *Adora2b*, *Acta2*, *Tgfb1*, *Il6*, *Fn1*, and *Gapdh*. (Table S4). *Gapdh* was employed as an internal control (normalizer) and dissociation curves were run to detect non-specific amplification. Relative gene expression levels were calculated after normalization with internal control *Gapdh* gene and specific primer pair efficiencies using the Pfaffl method [145].

### 4.14. Rat Lung Microarray Reanalysis (GSE15900)

To independently corroborate the A2BAR (*Adora2b*) mRNA expression data obtained by RT-qPCR (Section 2.1, Figure 1D) and to screen additional components of the adenosine axis, we reanalyzed a publicly available, diabetes-specific rat lung microarray dataset deposited in the NCBI Gene Expression Omnibus (GEO accession GSE15900) [48]. This dataset comprises whole-lung transcriptomic profiles from male Wistar rats, generated on the Affymetrix RAE230A GeneChip platform (12 animals total: *n* = 5 non-diabetic controls injected with citrate buffer vehicle, and *n* = 7 streptozotocin-induced diabetic rats [60 mg/kg, intraperitoneal], analyzed 4 weeks post-induction), with expression values normalized using the MAS5 algorithm. Differential expression analysis between control and diabetic groups was performed using GEO2R (National Center for Biotechnology Information), and normalized signal intensities for *Adora2b* (probe ID 1387395_at) and 22 additional adenosine axis-related transcripts—including the remaining adenosine receptors (*Adora1*, *Adora2a*, *Adora3*), ectonucleotidases of the CD39/CD73 family (Entpd1–6, Nt5e), nucleoside transporters (*Slc29a1–3*, *Slc28a1–3*), and adenosine-metabolizing enzymes (*Ada*, *Adk*, *Pnp*, *Nt5c2*, *Nt5c3a/b*, *Nt5m*)—were extracted for downstream analysis. Group distributions were assessed for normality using the Shapiro–Wilk test; given the presence of non-normal distributions, between-group comparisons were performed using the Mann–Whitney U test. Fold changes are expressed as log2(Diabetic/Control) of MAS5 signal intensity.

### 4.15. Public Single-Cell RNA-Seq Reanalysis

To evaluate the expression of *TGFB1*, *FN1*, and *ADORA2B* across human pulmonary cell populations, we performed a descriptive reanalysis of publicly available single-cell RNA-sequencing data using two independent, web-based interactive visualization platforms. Gene expression was queried in the dataset of Reyfman et al. [49], comprising single-cell transcriptomes from control and pulmonary fibrosis lung tissue, accessed through (i) the IPF Cell Atlas (www.ipfcellatlas.com), an interactive tool aggregating multiple published human lung fibrosis single-cell datasets, and (ii) a UCSC Cell Browser instance hosted by the Misharin laboratory at Northwestern University Feinberg School of Medicine (https://sqlifts.fsm.northwestern.edu/public/resources/ (accessed on 26 August 2026)), which provides cell type-resolved visualization of the same dataset. For each gene of interest, we recorded the percentage of cells expressing the transcript and/or mean expression level across annotated cell populations, stratified by disease status as defined in the original publication. Because these datasets derive from general pulmonary fibrosis (idiopathic pulmonary fibrosis and related interstitial lung disease) cohorts rather than diabetes-specific populations, this reanalysis was used as complementary translational evidence of cell type-specific expression patterns rather than direct validation of our diabetic lung disease model.

### 4.16. Statistical Analysis

Graphs and statistical analyses were performed using GraphPad Prism (version 11.0.0; Dotmatics, San Diego, CA, USA). Data are presented as mean ± standard deviation (SD), where *n* indicates the number of animals or the number of independent experimental replicates, as appropriate. Prior to inferential analyses, the normality of the data distribution was assessed for each group using the Shapiro–Wilk test (α = 0.05) (Table S5). For variables in which all groups fulfilled the normality assumption, one-way ANOVA followed by Tukey’s multiple comparison test was applied. For variables in which at least one group did not meet the normality assumption, the Kruskal–Wallis test followed by Dunn’s multiple comparison test was used. A *p*-value ≤ 0.05 was considered statistically significant. Given the ordinal nature and sample size of the inflammation grade data, pairwise comparisons were performed using the Mann–Whitney test with Bonferroni correction for multiple comparisons. Particular or additional statistical tests, when applied to specific analyses that deviate from this general framework (e.g., two-group comparisons, correlation analyses, or reanalysis of external datasets), are indicated in the corresponding subsection or figure legend.

## 5. Conclusions

This study provides experimental evidence that antagonism of the A2BAR with MRS1754 attenuates inflammation, fibrotic remodeling, and MΦ accumulation in the lungs of diabetic rats. Diabetic animals exhibited elevated extracellular adenosine and increased A2BAR protein expression, without a concomitant rise in *Adora2b* mRNA—a pattern independently corroborated by reanalysis of an unrelated diabetic rat lung microarray dataset and complemented by human single-cell transcriptomic data confirming *ADORA2B* expression across relevant pulmonary cell populations, including disease-associated epithelial and MΦ states in fibrotic lung. Diabetic animals also displayed marked pulmonary inflammation, including septal thickening and elevated IL-1β, IL-6, and TNF-α, and compartment-specific fibrotic remodeling with increased ECM and TGF-β1, α-SMA, and COL1A1 accumulation, alongside enhanced CD68^+^ MΦ infiltration in both lung tissue and BALF and a marked shift toward M1 and M2 polarization. MRS1754 treatment significantly attenuated these histological, molecular, and cellular alterations and fully normalized the M1/M2 MΦ balance to control levels, although MΦ infiltration and A2BAR protein expression were not completely reversed. BALF MΦ populations, particularly the CD163^+^ (M2) subset, correlated strongly with TGF-β1 and *Fn1* levels, and this association was further supported by human fibrotic lung reanalysis identifying MΦ as a major TGF-β1-expressing cell population, reinforcing MΦ as a central cellular link between diabetes-driven inflammation and fibrotic remodeling. In parallel, A2BAR blockade significantly increased ATP levels in BALF, accompanied by a non-significant trend toward reduced extracellular adenosine, consistent with changes in the extracellular purinergic milieu. Collectively, these findings indicate that pharmacological blockade of A2BAR attenuates diabetes-induced pulmonary inflammation, fibrosis, and MΦ accumulation, supporting a functional role for A2BAR in the pathogenesis of DLD, even though the precise intracellular signaling mechanism linking A2BAR engagement to these phenotypic outcomes remains to be fully defined (Figure 10). Although additional studies using type 2 diabetes models, functional respiratory assessments, further MΦ phenotyping, and extended mechanistic analyses are needed, our results highlight the potential of A2BAR antagonism as a promising therapeutic strategy to prevent or reduce pulmonary complications in DLD.

## Figures and Tables

**Table 1:** Summary of criteria evaluated to determine the degree of lung inflammation. The table shows the mean ± standard deviation (SD) score obtained in each parameter, the final score and the degree of lung inflammation. Criteria 1 to 5 were scored on a scale of 0 to 3.

Criterion Analyzed	Ctrl	DM	DM+MRS1754
1. Thickness of alveolar septa	0.40/3 (±0.64) ^†^	2.46/3 (±0.71) *	0.77/3 (±0.68) ^†^
2. Bronchiolar smooth muscle thickness	0.68/3 (±1.24) ^†^	2.58/3 (±0.86) *	0.25/3 (±0.65) ^†^
3. Infiltration of inflammatory cells	0.12/3 (±0.34) ^†^	2.43/3 (±0.59) *	0.88/3 (±0.63) *^,†^
4. Congestion in blood vessels	0.40/3 (±0.50) ^†^	2.53/3 (±0.55) *	0.97/3 (±0.78) *^,†^
5. Hyaline membrane formation	0.08/3 (±0.27) ^†^	2.02/3 (±1.03) *	0.48/3 (±0.74) ^†^
6. Final score	2/15 (±2.07) ^†^	12/15 (±2.57) *	3/15 (±1.94) ^†^
7. Degree of inflammation	absent	severe	absent

Control non-diabetic: Ctrl; diabetic: DM; diabetic MRS1754-treated (DM+MRS1754). Statistical analysis was performed using Kruskal–Wallis test followed by Dunn’s multiple comparison test. * *p* < 0.05 vs. Ctrl; ^†^ *p* < 0.05 vs. DM. *n* = 5 animals per group.

**Table 2:** Pearson correlation analysis between BAL macrophage marker expression (CD68^+^, CD163^+^, CD86^+^) and BAL TGF-β1 levels, pooled across all experimental groups.

Marker	Comparison	*n*	Pearson r	t Statistic	df	*p*-Value	Significance
Total CD68+ macrophages	CD68+ (%) vs. BAL TGF-β1	15	0.807	4.94	13	0.00027	***
CD163+ (M2) macrophages	CD163+ (%) vs. BAL TGF-β1	15	0.841	5.60	13	8.6 × 10^−5^	****
CD86+ (M1) macrophages	CD86+ (%) vs. BAL TGF-β1	15	0.662	3.18	13	0.00718	**

Abbreviations: BAL, bronchoalveolar lavage; TGF-β1, transforming growth factor beta 1; df, degrees of freedom. Significance: ** *p* < 0.01; *** *p* < 0.001; **** *p* < 0.0001.

**Table 3:** Pearson correlation analysis between BAL MΦ marker expression (CD68^+^, CD163^+^, CD86^+^) and whole-lung tissue *Fn1* mRNA levels, pooled across all experimental groups.

Marker	Comparison	*n*	Pearson r	t Statistic	df	*p*-Value	Significance
Total CD68+ macrophages	CD68+ (%) vs. tissue FN1 mRNA	15	0.718	3.72	13	0.00255	**
CD163+ (M2) macrophages	CD163+ (%) vs. tissue FN1 mRNA	15	0.714	3.67	13	0.00281	**
CD86+ (M1) macrophages	CD86+ (%) vs. tissue FN1 mRNA	15	0.573	2.52	13	0.02559	*

Abbreviations: df, degrees of freedom. Significance: * *p* < 0.05; ** *p* < 0.01. Note: CD68/CD163/CD86 were measured in BAL; *Fn1*/*Gapdh* mRNA was measured by RT-qPCR in whole-lung tissue, a different compartment from BAL.

## Data Availability

The minimal dataset supporting the findings of this study is provided as Supplementary Materials accompanying this article and has also been deposited in Mendeley Data at https://doi.org/10.17632/3tzmf8j496.2.

## Supplementary Materials

The following supporting information can be downloaded at: https://www.mdpi.com/article/10.3390/ijms27188171/s1.

## Abbreviations

The following abbreviations are used in this manuscript:

a.u.	Arbitrary unit
A2BAR	A2B Adenosine Receptor
AGE	Advanced Glycation End-products
ANOVA	Analysis of Variance
Akt1	AKT serine/threonine kinase 1
ATP	Adenosine Triphosphate
BAL	Bronchoalveolar Lavage
BALF	Bronchioalveolar Lavage Fluid
BSA	Bovine Serum Albumin
COL1A1	Collagen type 1 alpha 1
DLD	Diabetic Lung Disease
DM	Diabetes mellitus
ECM	Extracellular Matrix
ELISA	Enzyme-linked Immunosorbent Assay
FN	Fibronectin
GAPDH	Glyceraldehyde-3-phosphate dehydrogenase
ICC	Immunocytochemistry
H&E	Hematoxylin and Eosin
IDF	International Diabetes Federation
IHC	Immunohistochemistry
IL-1β	Interleukin-1β
IL-6	Interleukin-6
IPF	Idiopathic Pulmonary Fibrosis
iPSC	induced Pluripotent Stem Cell
JAK/STAT	Janus Kinase/Signal Transducer and Activator of Transcription
M1	M1 (Classically Activated) Macrophage
M2	M2 (Alternatively Activated) Macrophage
MTS	Masson’s trichrome stain
MΦ	Macrophage
NF-κB	Nuclear Factor Kappa-Light-Chain-Enhancer of Activated B Cells
PBS	Phosphate Buffered Saline
PB	Peribronchiolar
PFA	Paraformaldehyde
PI3K	Phosphoinositide 3-Kinase
RAGE	Receptor for AGE
ROS	Reactive Oxygen Species
RT-qPCR	Reverse Transcription quantitative Polymerase Chain Reaction
SbM	Submucosal
SD	Standard Deviation
SM	Smooth Muscle
SMAD3	SMAD Family Member 3
STZ	Streptozotocin
T2DM	Type 2 Diabetes Mellitus
TGF-β1	Transforming Growth Factor-β1
TNF-α	Tumor Necrosis Factor-α
WB	Western Blotting
α-SMA	α-Smooth Muscle Actin
