# Supplementary material for: A2BAR Antagonism Mitigates Pulmonary Inflammation and Fibrosis and Restores Macrophage M1/M2 Balance in Diabetic Rats
Source: Int J Mol Sci. 2026 Sep 14;27(18):8171. doi: 10.3390/ijms27188171 (PMC13607341; doi:10.3390/ijms27188171)
Supplement: Supplementary file 1 [file ijms-27-08171-s001.zip › Suppl Figures.zip!/Figure S1 - Torres-Arévalo.pdf]

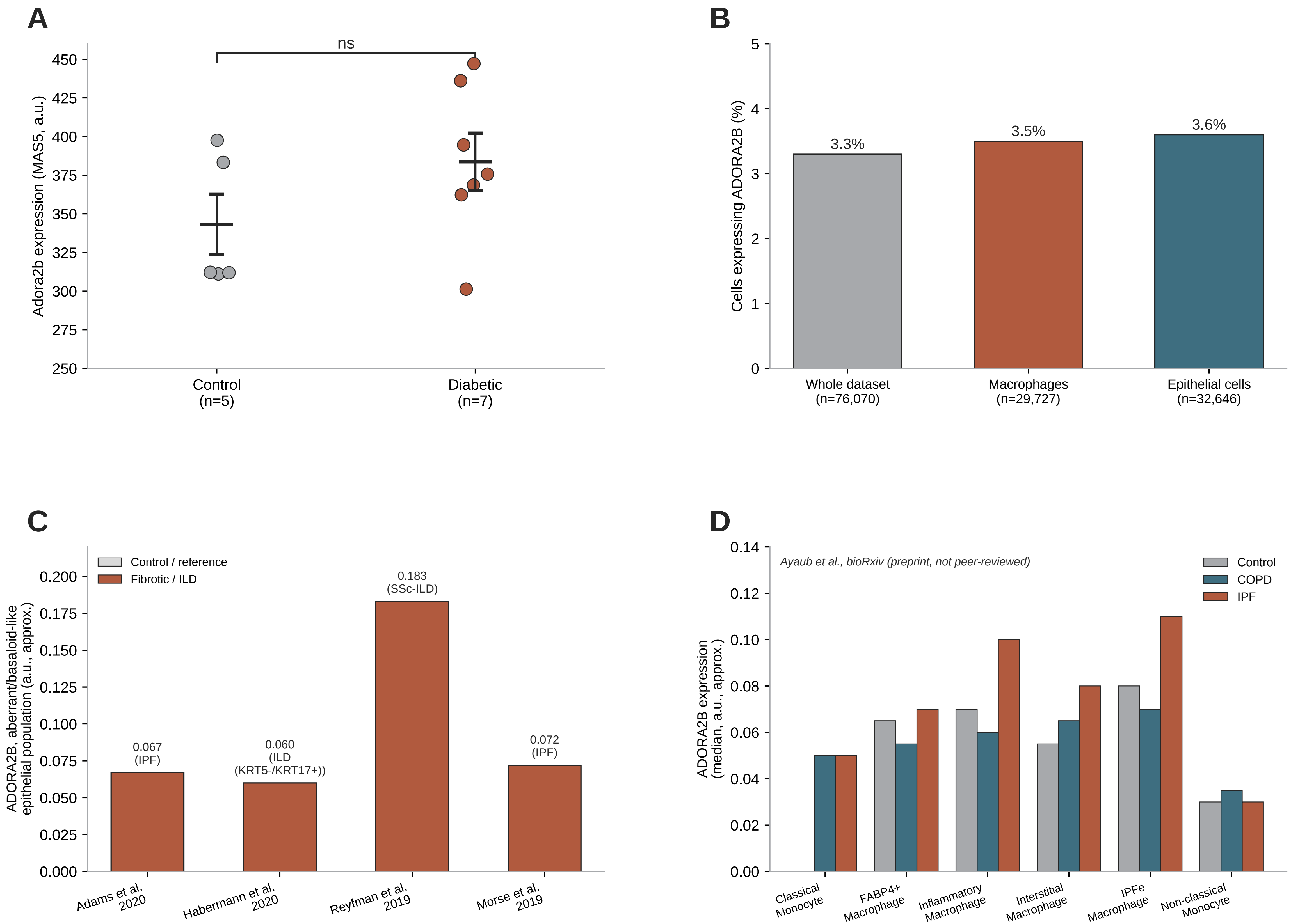

A
ns
Adora2b expression (MAS5, a.u.)
450
425
400
375
350
325
300
275
250
Control
(n=5)
Diabetic
(n=7)
B
Cells expressing ADORA2B (%)
5
4
3
2
1
0
3.3%
3.5%
3.6%
Whole dataset
(n=76,070)
Macrophages
(n=29,727)
Epithelial cells
(n=32,646)
C
Control / reference
Fibrotic / ILD
ADORA2B, aberrant/basaloid-like
epithelial population (a.u., approx.)
0.200
0.175
0.150
0.125
0.100
0.075
0.050
0.025
0.000
0.067
(IPF)
0.060
(ILD
(KRT5-/KRT17+))
0.183
(SSc-ILD)
0.072
(IPF)
Adams et al.
2020
Habermann et al.
2020
Reyfman et al.
2019
Morse et al.
2019
D
Ayaub et al., bioRxiv (preprint, not peer-reviewed)
Control
COPD
IPF
ADORA2B expression
(median, a.u., approx.)
0.14
0.12
0.10
0.08
0.06
0.04
0.02
0.00
Classical
Monocyte
FABP4+
Macrophage
Inflammatory
Macrophage
Interstitial
Macrophage
IPFe
Macrophage
Non-classical
Monocyte
