# Supplementary material for: A2BAR Antagonism Mitigates Pulmonary Inflammation and Fibrosis and Restores Macrophage M1/M2 Balance in Diabetic Rats
Source: Int J Mol Sci. 2026 Sep 14;27(18):8171. doi: 10.3390/ijms27188171 (PMC13607341; doi:10.3390/ijms27188171)
Supplement: Supplementary file 1 [file ijms-27-08171-s001.zip › Suppl Figures.zip!/Figure S10 - Torres-Arévalo.pdf]

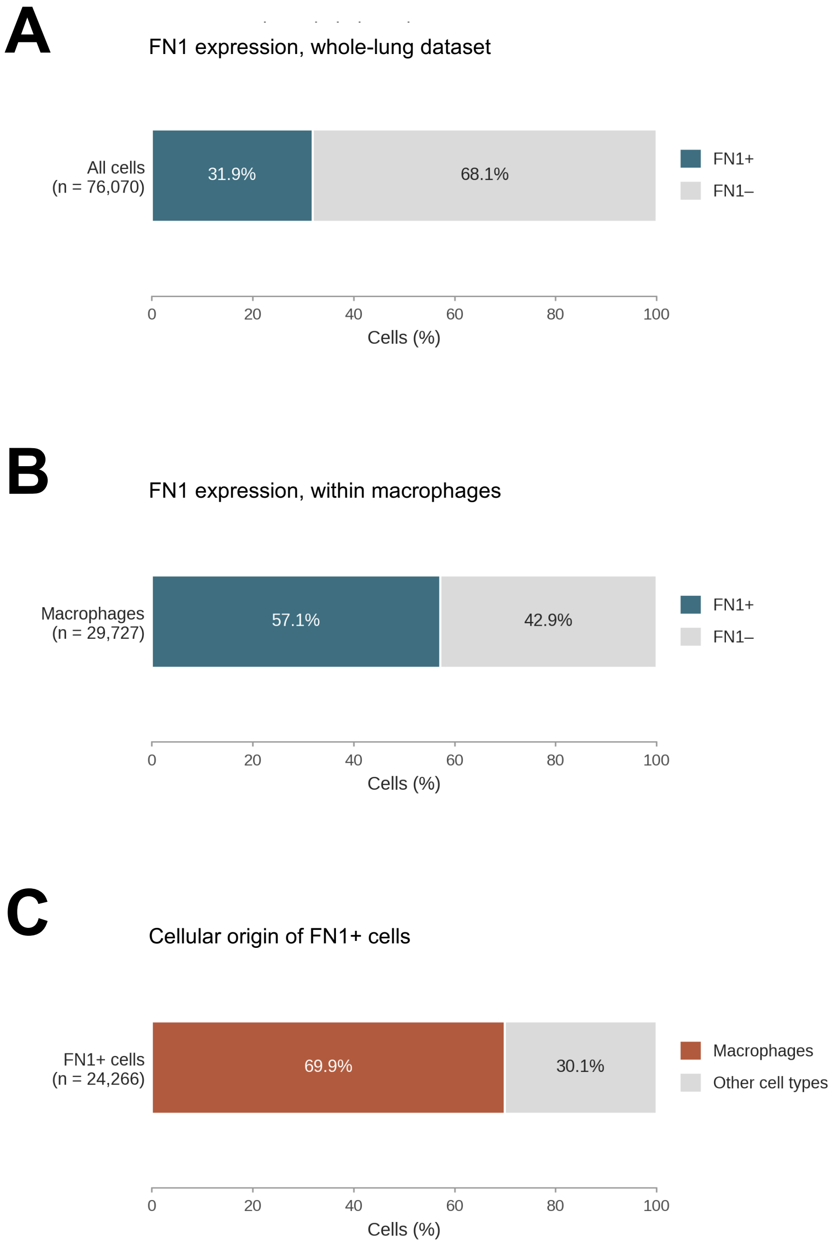
A
FN1 expression, whole-lung dataset
All cells
(n = 76,070)
31.9%
68.1%
FN1+
FN1–
0
20
40
60
80
100
Cells (%)
B
FN1 expression, within macrophages
Macrophages
(n = 29,727)
57.1%
42.9%
FN1+
FN1–
0
20
40
60
80
100
Cells (%)
C
Cellular origin of FN1+ cells
FN1+ cells
(n = 24,266)
69.9%
30.1%
Macrophages
Other cell types
0
20
40
60
80
100
Cells (%)
