# Supplementary material for: A2BAR Antagonism Mitigates Pulmonary Inflammation and Fibrosis and Restores Macrophage M1/M2 Balance in Diabetic Rats
Source: Int J Mol Sci. 2026 Sep 14;27(18):8171. doi: 10.3390/ijms27188171 (PMC13607341; doi:10.3390/ijms27188171)
Supplement: Supplementary file 1 [file ijms-27-08171-s001.zip › Suppl Figures.zip!/Figure S2 - Torres-Arévalo.pdf]

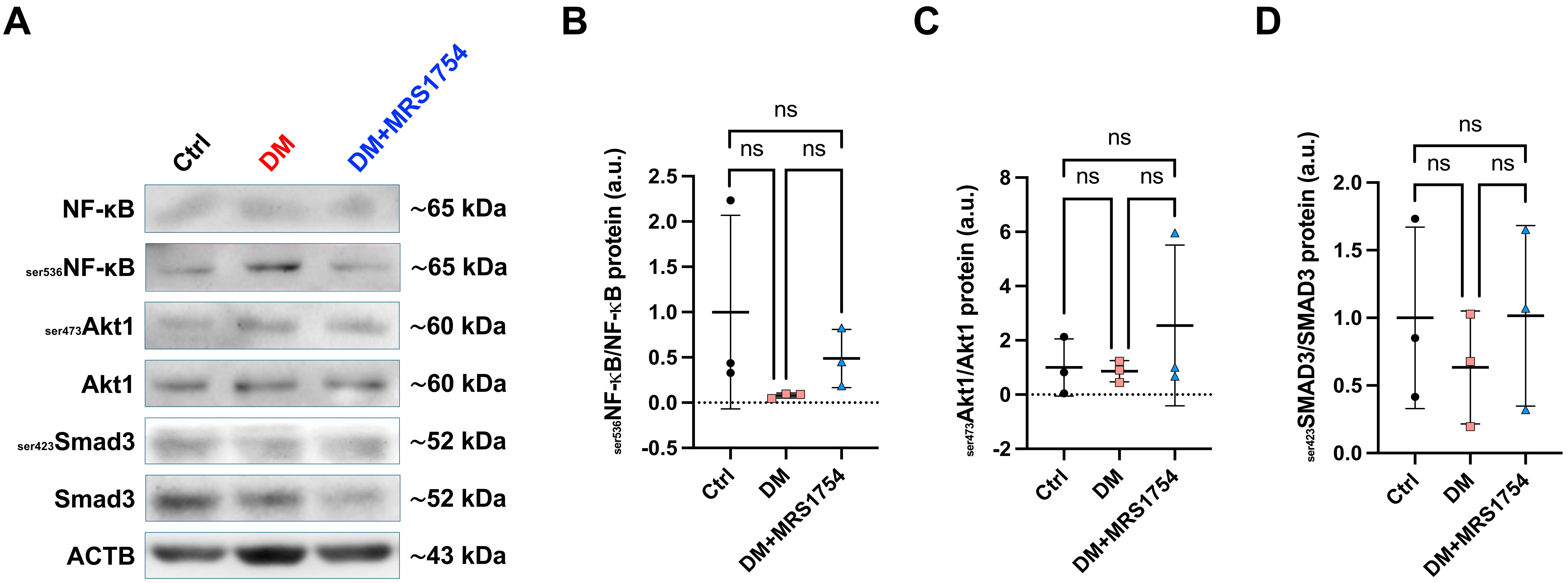
A
Ctrl
DM
DM+MRS1754
NF-κB
~65 kDa
ser536NF-κB
~65 kDa
ser473Akt1
~60 kDa
Akt1
~60 kDa
ser423Smad3
~52 kDa
Smad3
~52 kDa
ACTB
~43 kDa
B
ser536NF-κB/NF-κB protein (a.u.)
ns
ns
ns
2.5
2.0
1.5
1.0
0.5
0.0
-0.5
Ctrl
DM
DM+MRS1754
C
ser473Akt1/Akt1 protein (a.u.)
ns
ns
ns
8
6
4
2
0
-2
Ctrl
DM
DM+MRS1754
D
ser423SMAD3/SMAD3 protein (a.u.)
ns
ns
ns
2.0
1.5
1.0
0.5
0.0
Ctrl
DM
DM+MRS1754
