# Supplementary material for: A2BAR Antagonism Mitigates Pulmonary Inflammation and Fibrosis and Restores Macrophage M1/M2 Balance in Diabetic Rats
Source: Int J Mol Sci. 2026 Sep 14;27(18):8171. doi: 10.3390/ijms27188171 (PMC13607341; doi:10.3390/ijms27188171)
Supplement: Supplementary file 1 [file ijms-27-08171-s001.zip › Suppl Figures.zip!/Figure S3 - Torres-Arévalo.pdf]

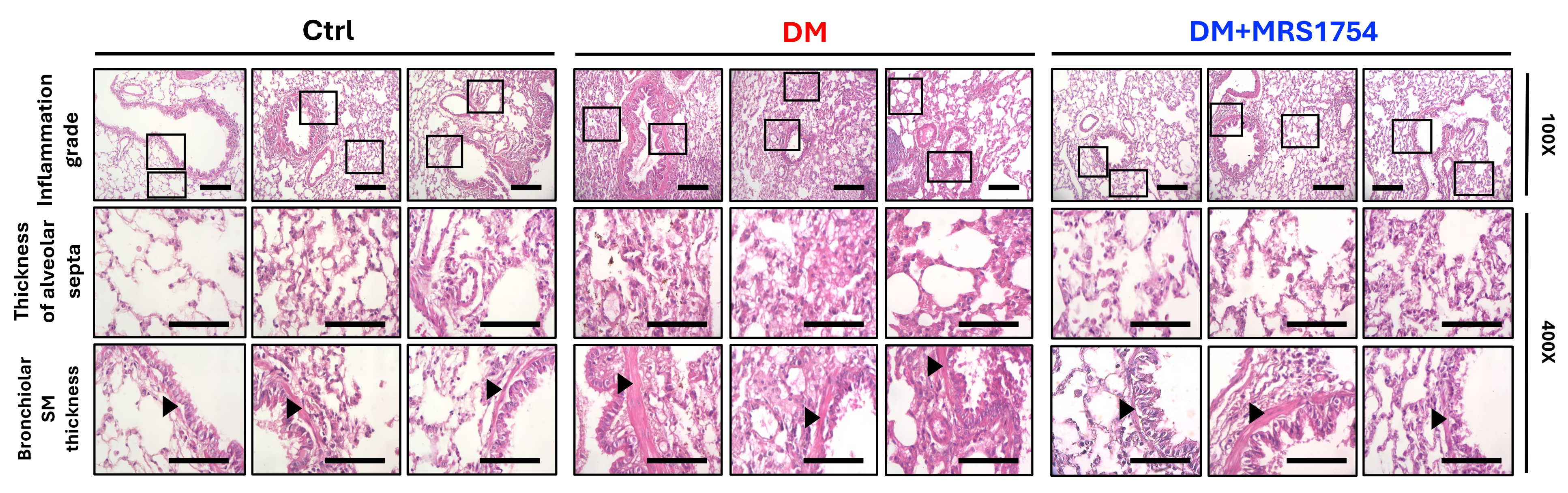
Ctrl
DM
DM+MRS1754
Inflammation grade
Thickness of alveolar septa
Bronchiolar SM thickness
100X
400X
