# Supplementary material for: A2BAR Antagonism Mitigates Pulmonary Inflammation and Fibrosis and Restores Macrophage M1/M2 Balance in Diabetic Rats
Source: Int J Mol Sci. 2026 Sep 14;27(18):8171. doi: 10.3390/ijms27188171 (PMC13607341; doi:10.3390/ijms27188171)
Supplement: Supplementary file 1 [file ijms-27-08171-s001.zip › Suppl Figures.zip!/Figure S8 - Torres-Arévalo.pdf]

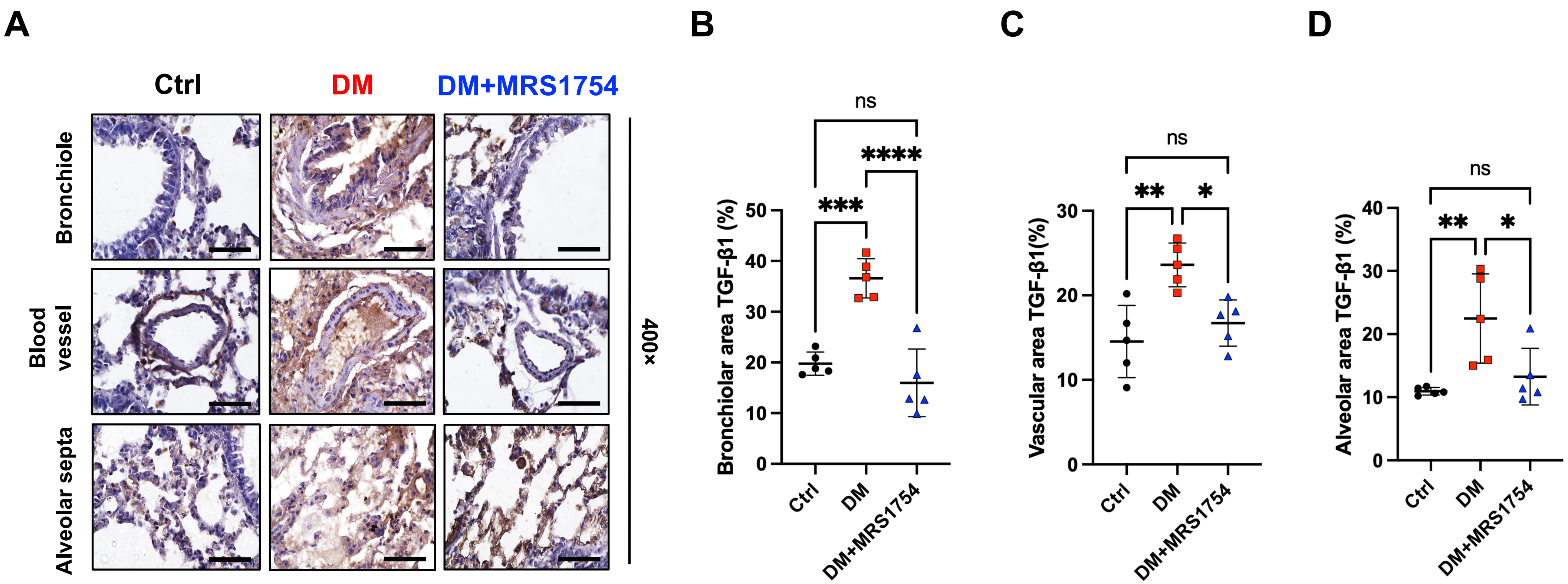
A
Ctrl
DM
DM+MRS1754
Bronchiole
Blood vessel
Alveolar septa
400×
B
Bronchiolar area TGF-β1 (%)
ns
***
****
Ctrl
DM
DM+MRS1754
C
Vascular area TGF-β1(%)
ns
**
*
Ctrl
DM
DM+MRS1754
D
Alveolar area TGF-β1 (%)
ns
**
*
Ctrl
DM
DM+MRS1754
