# Supplementary material for: A2BAR Antagonism Mitigates Pulmonary Inflammation and Fibrosis and Restores Macrophage M1/M2 Balance in Diabetic Rats
Source: Int J Mol Sci. 2026 Sep 14;27(18):8171. doi: 10.3390/ijms27188171 (PMC13607341; doi:10.3390/ijms27188171)
Supplement: Supplementary file 1 [file ijms-27-08171-s001.zip › Suppl Figures.zip!/Figure S9 - Torres-Arévalo.pdf]

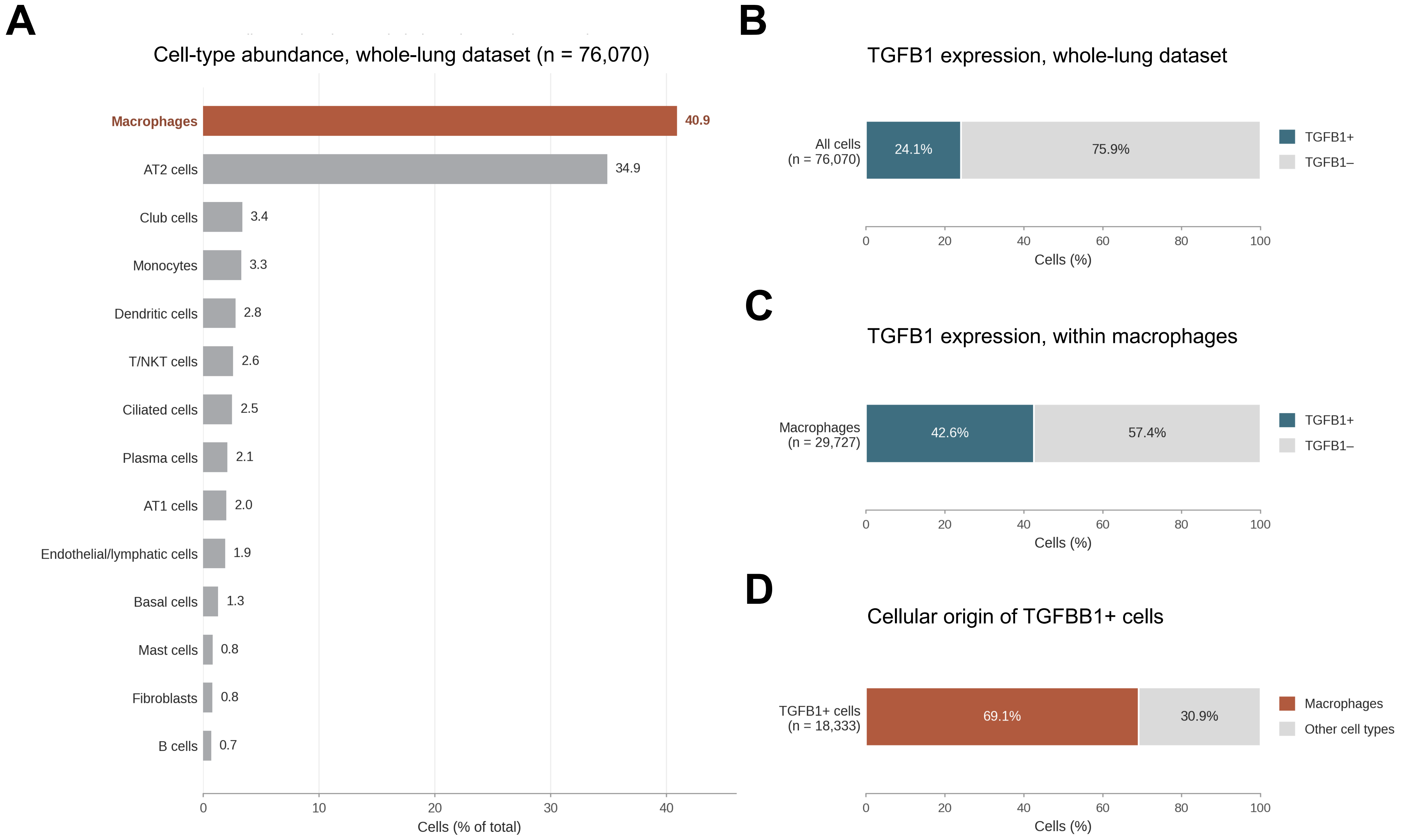

A
Cell-type abundance, whole-lung dataset (n = 76,070)
Macrophages
40.9
AT2 cells
34.9
Club cells
3.4
Monocytes
3.3
Dendritic cells
2.8
T/NKT cells
2.6
Ciliated cells
2.5
Plasma cells
2.1
AT1 cells
2.0
Endothelial/lymphatic cells
1.9
Basal cells
1.3
Mast cells
0.8
Fibroblasts
0.8
B cells
0.7
0
10
20
30
40
Cells (% of total)
B
TGFB1 expression, whole-lung dataset
All cells
(n = 76,070)
24.1%
75.9%
TGFB1+
TGFB1–
0
20
40
60
80
100
Cells (%)
C
TGFB1 expression, within macrophages
Macrophages
(n = 29,727)
42.6%
57.4%
TGFB1+
TGFB1–
0
20
40
60
80
100
Cells (%)
D
Cellular origin of TGFBB1+ cells
TGFB1+ cells
(n = 18,333)
69.1%
30.9%
Macrophages
Other cell types
0
20
40
60
80
100
Cells (%)
