# Supplementary material for: A2BAR Antagonism Mitigates Pulmonary Inflammation and Fibrosis and Restores Macrophage M1/M2 Balance in Diabetic Rats
Source: Int J Mol Sci. 2026 Sep 14;27(18):8171. doi: 10.3390/ijms27188171 (PMC13607341; doi:10.3390/ijms27188171)
Supplement: Supplementary file 1 [file ijms-27-08171-s001.zip › Suppl Figures.zip!/Supplementary Figure Captions - 15.07.2026.pdf]

Figure S1. A2BAR transcripts expression across independent rat and human lung datasets. (A) Adora2b mRNA expression (MAS5-normalized microarray intensity) in whole-lung tissue from control (n = 5) and streptozotocin-induced diabetic (n = 7) Wistar rats, reanalyzed from an independent, diabetes-specific dataset [49] (GEO GSE15900). Horizontal bars indicate mean ± SD; ns, not significant (Mann–Whitney U test, p = 0.43). (B) Percentage of cells expressing ADORA2B in the whole-lung dataset, macrophages, and epithelial cells from human lung single-cell RNA-seq data [50] (GEO GSE121611/GSE122960), accessed via the IPF Cell Atlas (www.ipfcellatlas.com) and a UCSC Cell Browser instance hosted by the Misharin laboratory (https://sqlifts.fsm.northwestern.edu/public/resources/). (C) ADORA2B expression in the disease-associated aberrant basaloid-like epithelial population (or its dataset-specific equivalent annotation: KRT5-/KRT17+, HAS1 High Fibroblasts) across four independent human single-cell RNA-seq studies of pulmonary fibrosis/interstitial lung disease [50–53], each showing expression largely restricted to fibrotic/ILD tissue with minimal or absent expression in the corresponding control/reference tissue. Values were estimated from publicly available interactive visualizations and should be interpreted as approximate. (D) ADORA2B expression (median, approximate) across monocyte and macrophage subpopulations in control, COPD, and IPF lungs, from a preliminary, not-yet-peer-reviewed dataset [54]; these data should be interpreted with appropriate caution pending formal peer review. Because the datasets in (B), (C), and (D) derive from general pulmonary fibrosis/ILD cohorts rather than diabetes-specific populations, they are presented as complementary translational evidence rather than direct validation of the diabetic lung disease model used in this study.

Figure S2. *In vivo* A2BAR blockade with MRS1754 and downstream signaling markers in the lungs of diabetic rats. (A) Representative Western blot images of ser536-phosphorylated NF-κB (~65 kDa), total NF-κB (~65 kDa), ser473-phosphorylated Akt1 (~60 kDa), total Akt1 (~60 kDa), ser423-phosphorylated SMAD3 (~52 kDa), total SMAD3 (~52 kDa), and ACTB (~43 kDa, loading control) in lung tissue from control non-diabetic (Ctrl), diabetic (DM), and diabetic MRS1754-treated (DM+MRS1754) rats. (B) Densitometric quantification of the ser536-NF-κB/NF-κB ratio, (C) ser473-Akt1/Akt1 ratio, and (D) ser423-SMAD3/SMAD3 ratio, expressed in arbitrary units (a.u.) and normalized to the Ctrl group. Each dot represents one animal (n = 3 animals per group). Graphs are presented as mean ± SD. Statistical analysis was performed using one-way ANOVA followed by Tukey's multiple comparison test. ns indicates no significant difference.

Figure S3. Impact of *in vivo* A2BAR antagonism on lung histopathological features associated with inflammation in diabetic rats. H&E-stained lung sections

from control non-diabetic (Ctrl), diabetic (DM), and diabetic MRS1754-treated (DM+MRS1754) rats are shown. Images acquired at 100× (scale bar = 200 μm) magnification provide an overview of inflammatory involvement within the pulmonary parenchyma. Higher-magnification micrographs (400×; scale bar = 100 μm) emphasize structural alterations, including changes in alveolar septal architecture, and bronchiolar smooth muscle (SM) thickening. Black arrowheads (►) denote bronchiolar SM.

Figure S4. Effect of *in vivo* MRS1754 treatment on IL-1β expression in lung tissue of diabetic rats. Representative lung sections from Ctrl, DM, and DM+MRS1754 rats were immunostained with anti-IL-1β antibody (A). Images illustrate bronchioles, blood vessels, and alveolar septa (magnification 400×; scale bar = 50 μm). Quantitative analysis shows the percentage of IL-1β–positive area in bronchioles (B), blood vessels (C), and alveolar septa (D). Seven non-overlapping fields per section were analyzed and averaged to obtain a single value per animal. Each dot represents one animal (n = 5 per group), and data are expressed as mean ± SD. Statistical analysis was performed using the Kruskal–Wallis test followed by Dunn's multiple comparison test for panels (B) and (C), and one-way ANOVA followed by Tukey's multiple comparison test for panel (D). Statistical significance is indicated as $^{*}p < 0.05$, $^{**}p < 0.01$, and $^{****}p < 0.0001$; ns indicates no significant difference.

Figure S5. Effect of *in vivo* A2BAR antagonism on TNF-*α* expression in lung tissue of diabetic rats. Representative lung sections from Ctrl, DM, and DM+MRS1754 rats were immunostained with anti-TNF-*α* antibody (A). Images illustrate bronchioles, blood vessels, and alveolar septa (magnification 400×, scale bar = 100 μm). Quantitative analysis shows the percentage of TNF-*α*–positive area in bronchioles (B), blood vessels (C), and alveolar septa (D). Seven non-overlapping fields per section were analyzed and averaged to obtain a single value per animal. Each dot represents one animal (n = 5 per group), and data are expressed as mean ± SD. Statistical analysis was performed using one-way ANOVA followed by Tukey's multiple comparison test. Statistical significance is indicated as $^{***}p < 0.001$, and $^{****}p < 0.0001$; ns indicates no significant difference.

Figure S6. Effect of *in vivo* MRS1754 treatment on extracellular matrix deposition in peribronchiolar and submucosal regions of bronchioles in diabetic rats. Representative Masson's trichrome–stained lung sections from control non-diabetic (Ctrl), diabetic (DM), and diabetic MRS1754-treated (DM+MRS1754) rats. Low-magnification images (100×, scale bar = 200 μm) show overall ECM distribution, while higher magnification (400×, scale bar = 100 μm) highlights structural details.

Insets delineate the peribronchiolar (PB; solid-line box) and submucosal (SbM; dashed-line box) compartments of the bronchioles.

Figure S7. Effect of *in vivo* MRS1754 treatment on COL1α1 expression in lung tissue of diabetic rats. Representative lung sections from Ctrl, DM, and DM+MRS1754 rats were immunostained with anti-COL1α1 antibody (A). Images illustrate bronchioles, blood vessels, and alveolar septa (magnification 400×, scale bar = 50 μm). Quantitative analysis shows the percentage of COL1α1–positive area in bronchioles (B), blood vessels (C), and alveolar septa (D). Seven non-overlapping fields per section were analyzed and averaged to obtain a single value per animal. Each dot represents one animal (n = 5 per group), and data are expressed as mean ± SD. Statistical analysis was performed using the Kruskal–Wallis test followed by Dunn's multiple comparison test. Statistical significance is indicated as $^{*}p < 0.05$, $^{**}p < 0.01$, and $^{***}p < 0.001$; ns indicates no significant difference.

Figure S8. Effect of *in vivo* A2BAR antagonism on TGF-β1 expression in lung tissue of diabetic rats. Representative lung sections from Ctrl, DM, and DM+MRS1754 rats were immunostained with anti-TGF-β1 antibody (A). Images illustrate bronchioles, blood vessels, and alveolar septa (magnification 400×, scale bar = 50 μm). Quantitative analysis shows the percentage of TGF-β1–positive area in bronchioles (B), blood vessels (C), and alveolar septa (D). Seven non-overlapping fields per section were analyzed and averaged to obtain a single value per animal. Each dot represents one animal (n = 5 per group), and data are expressed as mean ± SD. Statistical analysis was performed using the Kruskal–Wallis test followed by Dunn's multiple comparison test. Statistical significance is indicated as $^{*}p < 0.05$, $^{**}p < 0.01$, $^{***}p < 0.001$, and $^{****}p < 0.0001$; ns indicates no significant difference.

Figure S9. Macrophages are the most abundant and principal TGFB1-expressing cell population in the human fibrotic lung. Reanalysis of Reyfman et al. [50] (GEO GSE132771) using the IPF Cell Atlas. Panels A) and B) derive from the whole-lung, globally clustered dataset (Reyfman et al., their Fig. 1; n = 76,070 cells). Panels C) and D) combine this dataset with an independently, individually annotated macrophage reanalysis (Reyfman et al., their Fig. 5; n = 29,727 macrophages). As macrophage identity was defined using two distinct approaches across analyses, the estimate in panel D) (and the n = 12,664 TGFB1+ macrophage count used in panels C) and D) should be interpreted as approximate rather than as values directly reported by Reyfman et al. [50].

Figure S10. Macrophages are the most abundant and principal FN1-expressing cell population in the human fibrotic lung. Reanalysis of Reyfman et al. [50] (GEO

GSE132771) using the IPF Cell Atlas. Panels A) derive from the whole-lung, globally clustered dataset (Reyfman et al., their Fig. 1; n = 76,070 cells). Panels B) and C) combine this dataset with an independently, individually annotated macrophage reanalysis (Reyfman et al., their Fig. 5; n = 29,727 macrophages). As macrophage identity was defined using two distinct approaches across analyses, the estimate in panel C) (and the n = 16,974 FN1+ macrophage count used in panels B) and C) should be interpreted as approximate rather than as values directly reported by Reyfman et al. [50].

Figure S11. Pulmonary collagen deposition is established by one-month post-STZ injection and remains stable through three months. (A) Representative Masson trichrome-stained lung sections at each time point, shown at low magnification (40× and 100×, scale bar = 500 μm and 200 μm, respectively) (collagen-positive area in lung tissue, two representative regions per time point) and at higher magnification (400×, scale bar = 50 μm) for alveolar septa, bronchioles, and blood vessels, illustrating progressive peribronchial and perivascular collagen accumulation over the course of diabetes. (B) Collagen-positive area (Masson trichrome stain, % of total lung area) in non-diabetic controls (0) and at 1, 2, and 3 months post-STZ injection (n = 30 fields from n = 3 animals/group). Each point represents one histological field; horizontal bars indicate group means. Groups were compared using the Kruskal–Wallis test followed by Dunn's *post hoc* test with Bonferroni correction (***$p < 0.001$; ****$p < 0.0001$; ns, not significant).

Figure S12. Negative controls for immunohistochemical (IHC) staining. Representative images of lung sections processed with omission of the primary antibody (secondary antibody only) for (A) IL-1β (corresponding to Figure 3C), (B) TNF-α (Figure 3E), (C) COL1α1 (Figure 5E), (D) TGF-β1 (Figure 5G), and (E) CD68 (Figure 6A). Sections were otherwise processed identically to the corresponding immunostained sections, including secondary antibody incubation and chromogenic development, to confirm the absence of non-specific staining. Images were acquired at the same magnification and scale as the corresponding primary figure for each marker. No specific immunoreactivity was observed in the absence of primary antibody for any of the five markers evaluated.
