# Supplementary material for: A2BAR Antagonism Mitigates Pulmonary Inflammation and Fibrosis and Restores Macrophage M1/M2 Balance in Diabetic Rats
Source: Int J Mol Sci. 2026 Sep 14;27(18):8171. doi: 10.3390/ijms27188171 (PMC13607341; doi:10.3390/ijms27188171)
Supplement: Supplementary file 1 [file ijms-27-08171-s001.zip › Suppl Tables.zip!/Table S1 - Torres-Arévalo.pdf]

Table S1. Adenosine axis transcript screen in diabetic rat lung (GSE15900).

Category	Gene (probeset)	Probe ID	Control mean (MAS5, n=5)	Diabetic mean (MAS5, n=7)	log2FC (DM/Control)	*p*-value (Mann-Whitney U)
Receptors	Adora1	1370584_a_at	30.8	35.9	+0.22	0.432
Receptors	Adora2a	1368300_at	189.7	225.1	+0.25	0.268
Receptors	Adora2b	1387395_at	343.2	383.7	+0.16	0.432
Receptors	Adora3	1369272_at	61.6	60.0	-0.04	0.935
Ectonucleotidases	Entpd1 (CD39)	1368884_at	39.8	23.2	-0.78	0.149
Ectonucleotidases	Nt5e (CD73)	1369200_at	202.6	190.4	-0.09	0.073
Ectonucleotidases	Entpd2	1370845_at	126.2	117.4	-0.10	0.639
Ectonucleotidases	Entpd4	1374300_at	623.5	655.1	+0.07	0.876
Ectonucleotidases	Entpd5	1372774_at	330.4	295.0	-0.16	0.268
Ectonucleotidases	Entpd6	1368315_at	264.6	230.2	-0.20	0.432
Nucleoside transporters	Slc29a1 (ENT1)	1398295_at	3262.3	2800.7	-0.22	**0.048***
Nucleoside transporters	Slc29a2 (ENT2)	1376349_a_at	63.3	63.3	0.00	0.268
Nucleoside transporters	Slc29a3 (ENT3)	1374200_at	226.5	204.0	-0.15	0.268
Nucleoside transporters	Slc28a1 (CNT1)	1369250_at	6.1	14.9	+1.30	**0.030***
Nucleoside transporters	Slc28a2 (CNT2)	1368227_at	153.6	234.5	+0.61	0.073
Nucleoside transporters	Slc28a3 (CNT3)	1369447_at	288.8	140.0	-1.05	**0.003***
Metabolic enzymes	Ada	1370071_at	251.3	214.4	-0.23	0.268
Metabolic enzymes	Adk	1369630_at	2920.8	2285.1	-0.35	**0.030***
Metabolic enzymes	Pnp	1388337_at	5074.6	4579.9	-0.15	**0.048***

Category	Gene (probeset)	Probe ID	Control mean (MAS5, n=5)	Diabetic mean (MAS5, n=7)	log2FC (DM/Control)	*p*-value (Mann-Whitney U)
Metabolic enzymes	Nt5m	1375081_at	28.1	25.0	-0.16	0.343
Metabolic enzymes	Nt5c2	1391413_at	73.3	85.7	+0.23	0.343
Metabolic enzymes	Nt5c3a	1373412_at	505.4	543.8	+0.11	0.432
Metabolic enzymes	Nt5c3b	1389131_at	348.7	298.6	-0.22	0.073

Rat whole-lung microarray dataset GSE15900 (van Lunteren et al., 2014; Affymetrix RAE230A, MAS5-normalized) was reanalyzed to screen 23 transcripts of the adenosine axis (receptors, ectonucleotidases, nucleoside transporters, and adenosine-metabolizing enzymes) in Control (n = 5) versus streptozotocin-induced diabetic (n = 7) rat lung. Values are group means of MAS5 signal intensity (arbitrary units). log2FC was calculated as log2(Diabetic mean / Control mean). Group comparisons were performed using the Mann-Whitney U test. *$p < 0.05$, uncorrected for multiple comparisons (exploratory screen); no comparison survived Bonferroni correction ($\alpha = 0.05/23 \approx 0.0022$), although Slc28a3 (CNT3, p = 0.003) approached this threshold. For genes represented by more than one probeset on the array, the probeset with the lower p-value is shown.
