# Supplementary material for: A2BAR Antagonism Mitigates Pulmonary Inflammation and Fibrosis and Restores Macrophage M1/M2 Balance in Diabetic Rats
Source: Int J Mol Sci. 2026 Sep 14;27(18):8171. doi: 10.3390/ijms27188171 (PMC13607341; doi:10.3390/ijms27188171)
Supplement: Supplementary file 1 [file ijms-27-08171-s001.zip › Suppl Tables.zip!/Table S2 - Torres-Arévalo.pdf]

Table S2. Pathophysiological parameters in Sprague-Dawley rats.

	W1				W12			
Group	Body Weight (g)	Glycemia (mg/dl)	Proteinuria (mg)	Glucosuria (mg/dl)	Body Weight (g)	Glycemia (mg/dl)	Proteinuria (mg)	Glucosuria (mg/dl)
Ctrl	303 ± 32.5†	106 ± 28†	1.10 ± 0.25	0†	521 ± 24.5†	110 ± 34.7†	1.34 ± 0.58†	0†
DM	200 ± 32*	394 ± 193*	0.98 ± 0.41	687 ± 239*	198 ± 54.8*	554 ± 33*	5.89 ± 2.65*	1200 ± 447*
DM+MRS1754	205 ± 40*	345.6 ± 134.4*	1.56 ± 1.18	500 ± 0*	208 ± 49*	492 ± 21*	2.07 ± 0.95†	833 ± 258*

Abbreviations: Ctrl: Control non-diabetic; DM: diabetic; DM+MRS1754: diabetic treated with MRS1754; W1: week one; W12: week twelve.
* $p < 0.05$ vs Ctrl; † $p < 0.05$ vs DM. n = 10 animals per group.
