# Supplementary material for: A2BAR Antagonism Mitigates Pulmonary Inflammation and Fibrosis and Restores Macrophage M1/M2 Balance in Diabetic Rats
Source: Int J Mol Sci. 2026 Sep 14;27(18):8171. doi: 10.3390/ijms27188171 (PMC13607341; doi:10.3390/ijms27188171)
Supplement: Supplementary file 1 [file ijms-27-08171-s001.zip › Suppl Tables.zip!/Table S3 - Torres-Arévalo.pdf]

Table S3. List of primary and secondary antibodies.

Antibody	Catalog #	Host species	Company
anti-A2BAR	ab229671	Rabbit	Abcam, Cambridge, UK
anti-IL-1β	SC-52012	Mouse	Santa Cruz Biotechnology, Santa Cruz, CA, USA
anti-TNF-α	SC-52746	Mouse	Santa Cruz Biotechnology, Santa Cruz, CA, USA
anti-COL1A1	SC-293182	Mouse	Santa Cruz Biotechnology, Santa Cruz, CA, USA
anti-TGF-β1	SC-130348	Mouse	Santa Cruz Biotechnology, Santa Cruz, CA, USA
anti-CD86/B7-2	SC-28347	Mouse	Santa Cruz Biotechnology, Santa Cruz, CA, USA
anti-CD163	SC-58965	Mouse	Santa Cruz Biotechnology, Santa Cruz, CA, USA
anti-CD68	ab125212	Rabbit	Abcam, Cambridge, UK
anti-phospho NFkB p65 (Ser536)	3033	Rabbit	Cell Signaling Technology, Danvers, MA, USA
anti- NFkB p65	8242	Rabbit	Cell Signaling Technology, Danvers, MA, USA
anti-phospho-Akt 1 (Ser473)	9018	Rabbit	Cell Signaling Technology, Danvers, MA, USA
anti-Akt1	2938	Rabbit	Cell Signaling Technology, Danvers, MA, USA
anti-phospho-SMAD3 (Ser423/425)	9520	Rabbit	Cell Signaling Technology, Danvers, MA, USA
anti-SMAD3	9523	Rabbit	Cell Signaling Technology, Danvers, MA, USA
Peroxidase AffiniPure goat anti-rabbit IgG (H+L)	111-035-003		Jackson ImmunoResearch Lab, USA
anti-Rabbit IgG, Alexa Fluor™ Plus 488	A-11008	Goat	Thermo Fisher Scientific, Waltham, MA, USA
anti-Mouse IgG, Alexa Fluor™ Plus 568	A-11004	Goat	Thermo Fisher Scientific, Waltham, MA, USA
