# Supplementary material for: A2BAR Antagonism Mitigates Pulmonary Inflammation and Fibrosis and Restores Macrophage M1/M2 Balance in Diabetic Rats
Source: Int J Mol Sci. 2026 Sep 14;27(18):8171. doi: 10.3390/ijms27188171 (PMC13607341; doi:10.3390/ijms27188171)
Supplement: Supplementary file 1 [file ijms-27-08171-s001.zip › Suppl Tables.zip!/Table S4 - Torres-Arévalo.pdf]

**Table S4.** List of primers sequence used for RT-qPCR.

| Primer | Forward primer (Fw) | Reverse primer (Rv) |
|---|---|---|
| GAPDH | TCT CTG CTC CTC CCT GTT CTA | TAC GGC CAA ATC CGT TCA CA |
| A2BAR | AAG CTT ATG CAG CTA GAG ACG | GAT CCA AGC TCA GAC TGA AAG |
| IL-6 | ACT TCA CAA GTC GGA GGC TT | TGC CAT TGC ACA ACT CTT TTC |
| TGF-β | CTG CTG ACC CCC ACT GAT AC | AGC CCT GTA TTC CGT CTC CT |
| $\alpha$-SMA | ACC GGG AGA AAA TGA CCC AG | AGT CCA GCA CAA TAC CAG TTG T |
| FN1 | AGT GGG ATC GTC AGG GAG AA | AAC ACG TTG CTT CAT GGG GA |
