# Supplementary material for: A2BAR Antagonism Mitigates Pulmonary Inflammation and Fibrosis and Restores Macrophage M1/M2 Balance in Diabetic Rats
Source: Int J Mol Sci. 2026 Sep 14;27(18):8171. doi: 10.3390/ijms27188171 (PMC13607341; doi:10.3390/ijms27188171)
Supplement: Supplementary file 1 [file ijms-27-08171-s001.zip › Suppl Tables.zip!/Table S5 - Torres-Arévalo.pdf]

Table S5. Shapiro–Wilk normality test results and statistical test applied for each quantitative panel.

Figure	Variable	Ctrl (W / p)	DM (W / p)	DM+MRS1754 (W / p)	Normal?	Test applied
Fig 1B	ATP BALF	0.9953 / 0.9945	0.9139 / 0.4915	0.8570 / 0.2178	Yes	ANOVA + Tukey
Fig 1C	Adenosine BALF	0.8251 / 0.1277	0.8978 / 0.3979	0.7650 / 0.0408	No*	Kruskal-Wallis + Dunn
Fig 1D	A2BAR mRNA	0.9402 / 0.6670	0.8936 / 0.3753	0.8379 / 0.1591	Yes	ANOVA + Tukey
Fig 1F	A2BAR IHC	0.7204 / 0.0155	0.8837 / 0.3264	0.9324 / 0.6126	No*	Kruskal-Wallis + Dunn
Fig 2B	Inflammation grade	—	—	—	Ordinal	Mann-Whitney + Bonferroni
Fig 2C	Alveolar septa	0.9019 / 0.4204	0.7143 / 0.0134	0.9648 / 0.8411	No*	Kruskal-Wallis + Dunn
Fig 2D	SM thickness	0.9673 / 0.8574	0.9933 / 0.9898	0.8112 / 0.0996	Yes	ANOVA + Tukey
Fig 3A	IL-6 mRNA	0.9437 / 0.6919	0.9204 / 0.5326	0.9667 / 0.8535	Yes	ANOVA + Tukey
Fig 3B	TNF-α BALF	0.8473 / 0.1863	0.9433 / 0.6892	0.9186 / 0.5206	Yes	ANOVA + Tukey
Fig 3D	IL-1β IHC	0.8285 / 0.1355	0.8518 / 0.2001	0.8017 / 0.0836	Yes	ANOVA + Tukey
Fig 3F	TNF-α IHC	0.9242 / 0.5576	0.8217 / 0.1203	0.9394 / 0.6618	Yes	ANOVA + Tukey
Fig 4B	Alveolar MTS	0.9764 / 0.9144	0.8156 / 0.1079	0.9542 / 0.7669	Yes	ANOVA + Tukey
Fig 4D	PB MTS	0.9698 / 0.8739	0.9636 / 0.8329	0.9126 / 0.4835	Yes	ANOVA + Tukey
Fig 4E	SbM MTS	0.9700 / 0.8753	0.9554 / 0.7760	0.9222 / 0.5443	Yes	ANOVA + Tukey
Fig S4A	IL-1β bronchiolar	0.8082 / 0.0944	0.8840 / 0.3277	0.6778 / 0.0055	No*	Kruskal-Wallis + Dunn
Fig S4B	IL-1β vascular	0.7435 / 0.0259	0.8609 / 0.2314	0.7504 / 0.0300	No*	Kruskal-Wallis + Dunn
Fig S4C	IL-1β alveolar	0.9433 / 0.6891	0.9097 / 0.4658	0.7790 / 0.0540	Yes	ANOVA + Tukey
Fig S5A	TNF-α bronchiolar	0.8637 / 0.2418	0.9266 / 0.5737	0.8524 / 0.2022	Yes	ANOVA + Tukey
Fig S5B	TNF-α vascular	0.8943 / 0.3793	0.9420 / 0.6803	0.9587 / 0.7989	Yes	ANOVA + Tukey
Fig S5C	TNF-α alveolar	0.8845 / 0.3304	0.8615 / 0.2336	0.9281 / 0.5832	Yes	ANOVA + Tukey
Fig 5A	FN mRNA	0.9279 / 0.5819	0.9024 / 0.4236	0.9448 / 0.7002	Yes	ANOVA + Tukey
Fig 5B	α-SMA mRNA	0.9437 / 0.6919	0.9075 / 0.4525	0.8202 / 0.1171	Yes	ANOVA + Tukey
Fig 5C	TGF-β1 mRNA	0.9745 / 0.9035	0.9418 / 0.6790	0.9433 / 0.6895	Yes	ANOVA + Tukey
Fig 5D	TGF-β1 BALF ELISA	0.9475 / 0.7190	0.7938 / 0.0721	0.8523 / 0.2019	Yes	ANOVA + Tukey
Fig 5F	COL1α1 IHC	0.9521 / 0.7523	0.7886 / 0.0652	0.9182 / 0.5185	Yes	ANOVA + Tukey
Fig 5H	TGF-β1 IHC	0.9284 / 0.5853	0.9389 / 0.6585	0.9893 / 0.9773	Yes	ANOVA + Tukey
Fig S7A	COL1α1 bronchiolar	0.9567 / 0.7847	0.7072 / 0.0114	0.8890 / 0.3523	No*	Kruskal-Wallis + Dunn
Fig S7B	COL1α1 vascular	0.9062 / 0.4452	0.9111 / 0.4743	0.9570 / 0.7868	Yes	ANOVA + Tukey
Fig S7C	COL1α1 alveolar	0.9208 / 0.5348	0.9424 / 0.6828	0.8147 / 0.1062	Yes	ANOVA + Tukey
Fig S8A	TGF-β1 bronchiolar	0.9073 / 0.4513	0.9164 / 0.5070	0.8713 / 0.2716	Yes	ANOVA + Tukey
Fig S8B	TGF-β1 vascular	0.9956 / 0.9952	0.9719 / 0.8872	0.9541 / 0.7661	Yes	ANOVA + Tukey
Fig S8C	TGF-β1 alveolar	0.9621 / 0.8225	0.8806 / 0.3120	0.8130 / 0.1030	Yes	ANOVA + Tukey
Fig 6B	CD68 IHC	0.8218 / 0.1205	0.9365 / 0.6415	0.8326 / 0.1456	Yes	ANOVA + Tukey
Fig 6D	CD68 flow cytometry	0.8595 / 0.2264	0.9298 / 0.5948	0.9672 / 0.8573	Yes	ANOVA + Tukey
Fig 7B	$CD86^+$ (M1)	0.8769 / 0.2957	0.9379 / 0.6512	0.9813 / 0.9414	Yes	ANOVA + Tukey
Fig 7D	$CD163^+$ (M2)	0.8287 / 0.1359	0.8751 / 0.2879	0.9189 / 0.5228	Yes	ANOVA + Tukey

Abbreviations: Ctrl: control non-diabetic; DM: diabetic; DM+MRS1754: diabetic treated with MRS1754; W: Shapiro–Wilk statistic; p: Shapiro–Wilk *p* value.

*At least one group did not pass the Shapiro–Wilk normality test ($p < 0.05$); in these cases, the Kruskal–Wallis test followed by Dunn's multiple comparison test was applied instead of one-way ANOVA. For Figure 2B (ordinal histopathological score), pairwise comparisons were performed using the Mann–Whitney test with Bonferroni correction. n = 5 animals per group in all panels unless otherwise noted.
